# Investigative health and ecological risk assessment of trace elements in pharmaceutical deposition near Dhaka: An endemic industrial surge of Bangladesh

**DOI:** 10.1371/journal.pone.0338816

**Published:** 2026-01-05

**Authors:** Sharmin Akter Lisa, Sharmeen Nishat, Md. Kamal Hossain, Lokman Hosen, Mohammad Moniruzzaman, Md. Samrat Mohay Menul Islam, M. A. A. Shofi Uddin Sarkar

**Affiliations:** 1 Department of Chemistry, Bangladesh University of Engineering and Technology (BUET), Dhaka, Bangladesh; 2 Institute of Food Science and Technology, Bangladesh Council of Scientific and Industrial Research, Dhaka, Bangladesh; 3 Soil and Environment Research Section, BCSIR Dhaka Laboratories, Bangladesh Council of Scientific and Industrial Research, Dhaka, Bangladesh; 4 BCSIR Chattogram Laboratories, Bangladesh Council of Scientific and Industrial Research, Chattogram, Bangladesh; National College Autonomous, INDIA

## Abstract

The pharmaceutical industry (PMI) is one of the fastest-expanding sectors in Bangladesh. The environment of this country is suffering from pollution done by discharging PMI effluents into the surface water bodies. Twelve trace elements (As, Se, Pb, Be, Cd, Co, Cr, Cu, Mn, Ni, V, and Hg) were analyzed in water and sediment samples collected from nine distinct pharmaceutical outfalls using ICP-MS to monitor contamination from pharmaceutical waste. Pollution levels, associated health, and ecological risks were evaluated using various indicators. The water pollution indices depicted that the associated outfalls’ surface water was less polluted, and the health indices demonstrated that, although the risks were far below the threshold value, prolonged exposure to the metal content of the surface water, particularly Cd, may cause potential health hazards. The simulated probabilistic analysis estimated median carcinogenic risks for both adults and children below the 1 × 10^−4^ benchmark. According to Monte Carlo Simulation, about 0.5% of adults and 0.3% of children are at carcinogenic risk. The geoaccumulation index (I_geo_), enrichment factor (EF), and contamination factor (CF) values revealed that the study area was moderately contaminated with Cd, while other metal contamination was less prevalent. The potential ecological risk (PER) index revealed that the maximum studied locations were at low to moderate risk, whereas two locations were at considerable risk. Principal component analysis (PCA) reveals that, Se, Ni, and As had comparable environmental characteristics and made the largest contributions to the overall variance for sediment samples, whereas Ni, V, and As had similar environmental characteristics and made the largest contribution to the overall variation for water. Pearson correlation depicted a significant correlation among several elements, suggesting some anthropogenic activities contributed to contamination. Despite being categorized according to the USFDA, the cluster analysis revealed that the examined industries could be classified differently regarding trace elements release.

## Introduction

The pharmaceutical industry (PMI) is one of the most flourishing sectors in Bangladesh. The demand for pharmaceutical products has surged since Bangladesh has experienced an evolutionary development in the medical sector in recent years, which has led in emerging of several industries not only to fulfill the local demand but also to compete with the global market [1]. According to DGDA (Directorate General of Drug Administration), approximately 298 registered pharmaceutical manufacturers (PM) are prevailing in Bangladesh, of whom about 236 are in operation [2]. Despite having similarities in maintenance and housekeeping activities, the pharmaceutical industries vary in terms of waste stream production, the range of drug production, the sorts of production techniques employed, and the production scale. Consequently, the way that various industries contaminate the environment varies [3]. However, industrial advancement has raised intense concern about increasing environmental pollution [4]. Pharmaceutical wastewater contains toxic and hazardous substances, including antibiotics, steroids, metabolites, heavy metals or potentially toxic elements (PTEs), and other contaminants; most of which may be harmful to humans [5]. However, likewise, the pharmaceutical contaminants trace metals in pharmaceutical effluents should also be under consideration since metal compounds are vital ingredients for drug manufacturing. Potentially toxic elements (PTEs) have applications in the pharmaceutical industry for several reasons. Therefore, in addition to other organic contaminants, pharmaceutical effluents also contain trace elements in varied amounts [6,7]. Trace metal (TM) concentrations in surface water can increase due to the illicit and unregulated dumping of untreated industrial wastewater, municipal waste, and agricultural chemicals [8]. These detrimental metals have the proclivity to bioaccumulate in the food chain and cause catastrophic harm to the ecosystem when concentrations are above the maximum permissible levels [9,10]. Adsorption, hydrolysis, and precipitation of these metals result in the production of free metal ions, some of which are deliquesced in water, and a sizeable fraction of which accumulate in the undermost sediments, resulting in the generation of ecological risk [11]. The German Water Research Institute, IWW, analyzed 123,761 cases worldwide and detected 559 pharmaceuticals and their derivatives. This included 38 unique medications present in groundwater, surface water, and drinking water, as well as in effluent, influent, metabolites, and sludge from wastewater treatment facilities [12]. According to the USFDA (United States Food and Drug Administration), the active pharmaceutical ingredients should not exceed 1 µg/L in effluent [13]. Inappropriately treated wastewater can contaminate water bodies and subsequent sediments, which ultimately creates ecological concern [14]. The acute toxicity of pharmaceutical chemicals, including genotoxicity and mutagenic potential, has recently gained significant attention, resulting in several studies on the effects of pharmaceutical effluents on the ecosystem and the consequential human wellness [5]. The European Union’s priority substances instruction from the year 2014 emphasizes the need to address the potentially harmful effects of pharmaceuticals [12]. Hence, an exploratory risk evaluation of trace metals(loids) in growing PMI will open a new era in environmental sustainability research. Even though numerous studies have been conducted on trace element contamination due to the development of other industrial sectors, surprisingly, very few studies have been conducted on pharmaceutical industrial regions [5,15]. To the best of our knowledge, no systematic research on the area we selected for our study has yet been done on toxic elements in pharmaceutical discharge. Even though very few researchers have reported toxic element profiles in pharmaceutical effluent in Bangladesh, they have analyzed toxic elements using conventional analytical techniques (high-performance liquid chromatography and atomic absorption spectroscopy) and assessed the ecological risk only. Considering the aforementioned facts, water and sediment samples were collected from nine distinct locations in the current study. The locations were classified into three groups based on the types of pharmaceutical industries. And we have determined the potentially toxic element level utilizing the highly advanced ICP-MS (inductively coupled plasma mass spectrometry) technique and investigated both ecological and human health risks simultaneously for better visualization of detrimental effects of toxic metals. Which has made this research unique. As the combined analysis of water and sediment reveals a better understanding of contamination patterns and is relatively unexplored in the context of Bangladesh’s pharmaceutical industries so, the primary objectives of this research were to determine the quality parameters of water from the pharmaceutical outfalls and to quantify the perilous elements in the contaminated water and corresponding sediments to conduct a health risk assessment, encompassing both carcinogenic and non-carcinogenic risk based on US Environmental Protection Agency (USEPA) guidelines [16], as well as to assess the ecological risks using geochemical approaches. Optimistically, these research findings are expected to contribute to assessing the detrimental health and ecological effects due to trace element-rich pharmaceutical discharge and identifying the sources and origins of trace element contamination. The assessment of the indices was influenced by the necessity to extensively investigate both the chemical and ecological impacts of pharmaceutical effluents in terms of trace elements. Hence, this research is focusing on the repercussions of pharmaceutical effluents on both human health and the ecosystem, thus setting a paradigm for future studies with other pollutants of pharmaceutical industries.

## Materials and methods

### Study area

**Fig 1:**
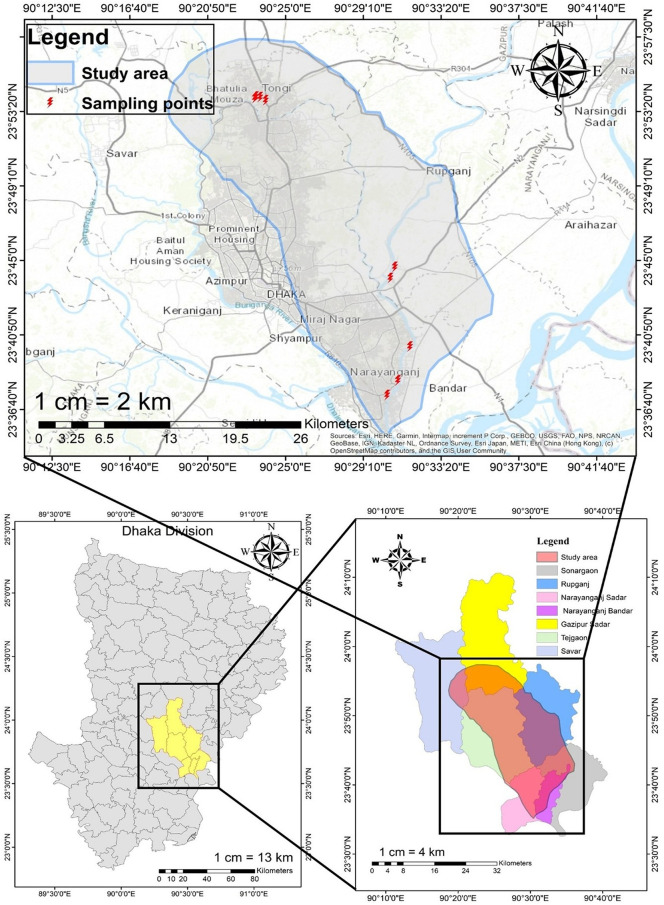
Location of the sampling sites at Gazipur and Narayanganj. The map was formed by ArcGIS software of version 10.8.

Nine PMI regions in Gazipur and Narayanganj, which are situated in the northeast and southeast part of Dhaka district, respectively, were selected as study areas (Fig 1). Water and sediment samples of three location points were collected from these nine regions (total of 27 water samples and 27 sediment samples) where wastewater is discharged particularly from pharmaceutical industries during the afternoon in September of 2022. Random sampling was employed to provide an unbiased representation of pharmaceutical effluent contamination across the study area, enabling a reliable estimate despite potential spatial heterogeneity. PMI was grouped into three types: one type comprises A1, A2, A3, and A4 location points, where outfalls are from four USFDA-approved and the official ERA (Environmental Risk Assessment) authority-approved pharmaceutical industries [17]. The second type consists of B1, B2, B3, and B4 location points, where outflows are from four middle-class (not approved by USFDA yet, but recently rising industries in Bangladesh) pharmaceutical industries, and the third type is location point C, which is an integrated outfall where four USFDA-approved industries discharge their wastewater. There is one polymer factory and two knits apparels near sample locations A1, A2, B1, and C, one knit apparel near sample locations A3 and B2, one dockyard and one power plant near sample location A4, two melamine factories, one sweater factory, and one warehouse near sample location B3 and no other industry near sample location B4. Fig 1 shows the location of the sampling sites, and the latitudes and longitudes of the sampling sites are detailed in S1 Table. The Turag River originates from the Bangshi River and joins the Buriganga at Mirpur in Dhaka. Similarly, the Shitalakhya River, which branches off the Old Brahmaputra, flows through the eastern region of Dhaka District, almost paralleling the Old Brahmaputra, passing through Narayanganj before joining the Dhaleshwari River. The small water bodies into which the industries throw their emissions were directly connected to the Turag and Shitalakhya rivers. The areas are densely populated, and the population depends on the water bodies for their domestic use [18].

### Sample collection

A random sampling strategy has been adopted for this study. Within a 5-meter radius of a sample collection site, nine location points were sampled for water and sediment, with triplicate of each point. The water samples were immediately stored in 100 mL propylene bottles that had been pre-sterilized with 20% HNO_3_ and then rinsed with distilled water. The air-dried bottles were rinsed three times with the sample water before sample storage. As soon as possible, the obtained samples were appropriately labeled and stored in an icebox. The final elute was prepared for analysis in the laboratory by filtering the water samples using a 0.45 µm filter paper to separate the suspended solids at first and then acidified with pure nitric acid. The sediment samples were collected from the sampling sites at a depth of 0.25–1 m using a plastic spatula. Triplicate sediment samples were collected within a 5 m circle of the sample collection. After being temporarily preserved in an ice-filled cooler box, the sediment samples were placed in acid-washed polyethylene bags and brought to the Bangladesh Council of Scientific and Industrial Research’s Soil and Environment laboratory in Dhaka. A porcelain mortar and pestle were used to grind the sediment samples uniformly after they had been allowed to air dry. To maintain homogeneity, the ground samples were sieved through a 2 mm mesh and then sealed in clean ziplock plastic bags to await additional analysis.

### Sample preparation

The water and sediment samples were prepared for analysis following the method described by Hasan *et al.* (2021) and Shorna *et al.* (2021), respectively [8,19]. Water samples were prepared for analysis in the laboratory by filtering the water samples using 0.45 µm filter paper to separate the suspended solid at first. Typically, 50 mL of water sample was taken in an acid-washed pyrex beaker and 10 mL of pure nitric acid (HNO_3_) was added to it. The volume of the mixture was lowered to almost 5–10 mL by digesting at 80 °C on a hot plate. The heating and addition of concentrated HNO_3_ were continued until a light-colored clear solution was obtained. Afterwards, the samples were cooled, and then filtered with Whatman no. 41 filter paper. The volume of the filtrate was adjusted to 50 mL with deionized water and stored for further analysis. Continually, 1 g of sediment sample was pre-digested overnight in 10 mL of nitric acid. The mixture was then left to heat at 100–180 °C for 5–6 h on a hot plate until the solution became clear. 5 mL of perchloric acid was added for complete digestion and further heated until the volume decreased. Just like water samples, it was then filtered followed by volume up to 50 mL. Blank samples were also prepared following the same procedure stated above.

### Chemicals and reagents

Supelco’s multi-element standard solution XIII was utilized for the ICP-MS analysis of trace elements. Throughout the experiment, deionized water was used for all solution preparation and dilution. Analytical-grade Merck, Germany, reagents were utilized for sample digestion, and metal content blank levels were verified to ensure the absence of target elements.

### Instrumentation

A multiparameter water analyzer (HQ 2200 portable multimeter) was used to measure the physical parameters, including pH, temperature, electrical conductivity, total dissolved solids, and dissolved oxygen (DO) of the water samples. Chemical oxygen demand (COD) was analyzed by USEPA-approved reactor digestion method using a HACH DR 3900 spectrophotometer that is appropriate for the determination of both high range (20–1500 mg/L) and low range (3–150 mg/L).

The concentrations of As, Se, Mn, Pb, Cu, Be, Cd, Cr, Ni, Co, V, and Hg in the water and sediment samples were determined using an inductively coupled plasma mass spectrometer (ICP-MS), NexION 2000, Perkin Elmer, USA. S2 Table lists each element’s limit of quantification (LOQ) and limit of detection (LOD). To calculate concentrations, calibration curves with a correlation coefficient (R2) value higher than 0.999 were chosen. The accuracy of the dataset was confirmed using certified reference materials obtained from Sigma-Aldrich, Germany. Calibration using internal standards was used to determine the trace element levels.

### Ethical statement

Formal authorization was not required to conduct the investigation in the manuscript. Samples were collected without disruption to the local community and damage to the surrounding environment. The entire experimental procedures were safe and non-invasive.

#### Inclusivity in global research.

Additional information regarding the ethical, cultural, and scientific considerations specific to inclusivity in global research is included in S1 File.

### Quality control and assurance

To assure the accuracy, sensitivity, and precision of the analytical procedure of ICP-MS, sediment standard reference (Stream Sediment Reference Material, GBW07309) and natural water reference (NIST-SRM-1640) purchased from Sigma-Aldrich were analyzed as certified reference material. The concentration of elements for GBW07309 and NIST-SRM-1640 was within the certified range. The analytical precision of the replicate was within 10% variability. Each sample was measured in triplicate to minimize the errors. Calibrated standards were analyzed further after every five elements to check their quality for quality control. The data on quality control have been given in S3 and S4 Table.

### Water pollution status and health risk assessment of metalloid)s in water

Water pollution indices- Heavy metal pollution index (HPI), Heavy metal evaluation index (HEI), Degree of contamination (C_d_), and related health risks (Hazard Quotient, Hazard Index, and Total Carcinogenic risk were calculated following different equations which are mentioned in the S1 file.

### Ecological risk assessment from soil contamination

Concentrations of PTEs were compared to the average shale value (ASV) of elements in the Earth’s crust mentioned by Turekian and Wedepohl (1961), toxicological reference value (TRV), and threshold effect level (TEL) proposed by USEPA (USEPA, 1999; 2002). As Average Shale Value (ASV) refers to the average concentration of chemical elements found in finely grained sedimentary rock that is considered a representative sample of the Earth’s upper continental crust, comparison with this value gives information about the amount of any element that is elevated in the study area. TEL shows whether or not adverse biological effects at the measured PTE level are expected, whereas TRV values are used to evaluate the possible dangers of environmental pollutants to human health. Soil pollution indices – Geoaccumulation index (I_geo_), Enrichment Factor (EF), Contamination Factor (CF), Pollution load index (PLI), and Potential Ecological Risk (PER) were also calculated to measure the ecological risk following some equations mentioned in S1 File.

### Statistical analysis

The mean ± standard deviations and other calculations and graphs of the trace element concentrations in water and sediment samples were determined by Microsoft Excel 2016 and Origin Pro 2024. The Pearson correlation matrix, principal component analysis (PCA), and cluster analysis were done using the statistical package SPSS (IBM SPSS 25) and Origin Pro 2024 software. The Monte Carlo simulation was done using RStudio. Variability in human vulnerability, environmental conditions, and methodological limitations make risk assessment inherently uncertain [20–22]. This was addressed by estimating the probabilistic carcinogenic risk associated with total cancer risk (TCR) by a Monte Carlo simulation using RStudio. This method offers a strong probabilistic risk distribution by allowing for the inclusion of variability and uncertainty in input parameters.

Applying the same exposure and toxicity criteria outlined in the Health Risk Analysis, Monte Carlo simulation was run using the mean total cancer risk (TCR) from all metals for adults and children. Following accepted risk assessment procedures, the simulation used a single-point deposition approach with the mean TCR and standard deviation (SD) as input parameters. To attain numerical stability, 10,000 iterations were performed, and the 5^th^, 50^th^, and 95^th^ percentiles represented the range of possible risk outcomes. This approach guarantees statistically sound and transparent results by adhering to best practices in probabilistic risk assessment [23]. The simulation reduces the possibility of overestimation or underestimation by providing a variety of realistic risk estimations, providing a nuanced understanding of carcinogenic risk.

## Results and discussion

### Physicochemical parameters of the water samples

Physicochemical parameters of the water samples, like water temperature, pH, total dissolved solids (TDS), electrical conductivity (EC), chemical oxygen demand (COD), and dissolved oxygen (DO), were analyzed, which are described in S5 Table.

The pH values of the water samples ranged from 7.80 to 8.57. The highest pH value was observed in region B1 (8.57), and the lowest was in B4 (7.8). Since pH is one of the most widely used measuring criteria for most types of water, it has an impact on both biological and chemical reactions [24]. According to ECR (Environment Conservation Rules) Bangladesh (2023), the pH of surface water should be 6.5–8.5 [25]. All the samples had pH within the range, except B1. Ponmurugan and Rangasamy, Mayabhate *et al,* and Rao *et al* found the pH of pharmaceutical industry wastewater to be 6.01 [26], 6.5–7.0 [27] and 7.5 [28] respectively. The TDS values of the water samples ranged from 105.4 to 596 mg/L, which are far below the ECR standard. TDS was found in pharmaceutical wastewater, 622 mg/L and 20,000 mg/L in Ponmurugan and Rangasamy, and Rao *et al,* respectively [26,28]. EC expresses the presence of excess ions in contaminated water [24]. Therefore, it has a strong relation with TDS. In this study, the highest EC was obtained in B2 (1201.42 µS), and the lowest EC was in B4 (211.04). DO is known to influence aquatic life. According to ECR, the DO of any surface water should be greater than 5. In the present study, only A2, A3, and C sampling sites had the desired DO values (7.98, 6.65, and 7.03), whereas the other sampling sites had lower DO. This indicates that all but three (A2, A3, and C) of the chosen sites’ water samples are contaminated and unsuitable for aquatic life. COD is one of the important parameters to determine the quality of chemically oxidizing matter. No sampling sites exceeded the permissible limit of COD by ECR, where B4 had the lowest COD (4.2 mg/L) and A4 had the highest COD (73.6 mg/L) value. COD of effluent water from pharmaceuticals found 548 mg/L, 2000–3000, and 25000 in Ponmurugan and Rangasamy; Mayabhate *et al* and Rao *et al* [26–28]. The temperature of all the sampling sites was higher than 25ºC C which is the maximum allowable limit by ECR for surface water. It is possibly due to the location of the sampling sites since the water samples were taken directly from the outflows of the industries. Therefore, the physicochemical parameters of the studied water samples were analogous to the limit specified by ECR, as well as the values mentioned in earlier studies, probably due to the wide range of treatment methods implemented by all pharmaceutical industries [29].

### Trace element concentration in water samples

**Fig 2:**
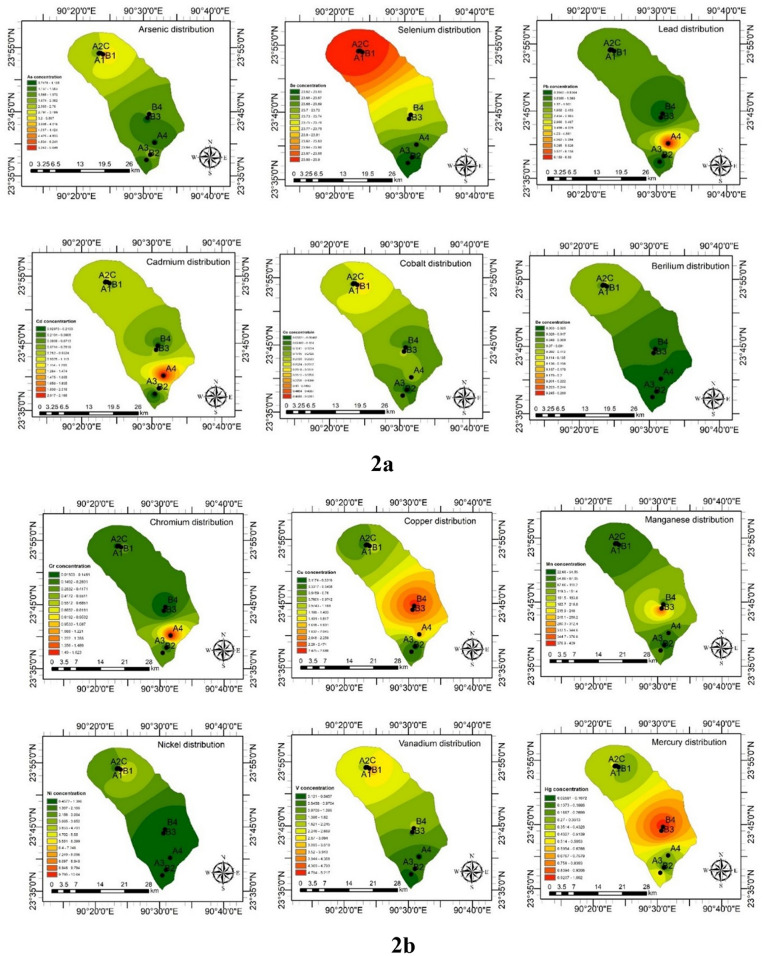
Spatial distribution of trace elements a. (As, Se, Pb, Cd, Co, Be); b. (Cr, Cu, Mn, Ni, V, Hg) in surface water of the nine location points. The map was formed by ArcGIS software of version 10.5.

The trace element concentration in the water samples collected from the pharmaceutical outfalls is demonstrated in Table 1. The average value of twelve trace elements followed the decreasing trend of Mn> As> Ni > V > Pb > Cu > Cd > Hg > Cr > Co > Se > Be, but differences in this trend were found in individual sample sites. The trace element concentrations found in the present study were likened to the permissible limit of ECR, Bangladesh (2023) for industrial effluents into surface water [25], USEPA (2018) [30], and WHO for drinking water (2023) [31]. As per Table 1, all the elements are far below the permissible limits for industrial effluents and outfalls declared in ECR, 2023, which makes a positive sense about the pharmaceutical industries of Bangladesh. The pharmaceutical business has recently become one of Bangladesh’s fastest-growing economic sectors, and to compete with global brands like Pfizer, AstraZeneca, Sanofi, and others for worldwide market share, they are compelled to adhere to USFDA regulations [17]. The USFDA made strict regulations for maintaining environmental protection. Compared with the WHO and USEPA drinking water limits, the trace elements were found within the limits in almost all the water samples except Mn in the B3 sampling site (409.94 µg/L). The results were low, possibly due to the proper effluent treatment of the PMIs. The concentration of Mn in B3 was found to be slightly higher than the drinking water quality limit by the WHO. An increased amount of Mn in the water bodies may come from nearby power generation plants and similar types of industries [19] or from geogenic origin and anthropogenic sources [32]. The spatial distribution of trace element content in water was revealed in Fig 2, from which it can be seen that the southeastern side of the study area is highly concentrated with Hg, Cu, Cd, and Pb, and the northeastern part is moderately concentrated with As, Se, Co, and V.

**Table 1:** Trace element concentration (Mean ± SD) in the water samples (in µg/L).

Sampling sites	As	Se	Pb	Be	Cd	Co	Cr	Cu	Mn	Ni	V	Hg
A1	1.633 ± 0.008	0.273 ± 0.003	0.935 ± 0.005	0.011 ± 0.004	0.705 ± 0.009	0.31 ± 0.008	0.139 ± 0.004	0.197 ± 0.002	113.57 ± 0.006	1.237 ± 0.009	1.509 ± 0.006	0.762 ± 0.006
A2	0.995 ± 0.005	0.172 ± 0.008	1.911 ± 0.005	0.029 ± 0.009	0.546 ± 0.007	0.099 ± 0.003	0.055 ± 0.004	0.08 ± 0.004	20.535 ± 0.005	0.419 ± 0.005	1.458 ± 0.004	0.322 ± 0.005
A3	2.49 ± 0.006	0.129 ± 0.009	0.826 ± 0.006	0.005 ± 0.005	0.811 ± 0.008	0.055 ± 0.008	0.083 ± 0.005	0.208 ± 0.005	27.54 ± 0.007	0.456 ± 0.008	1.05 ± 0.004	0.088 ± 0.004
A4	1.073 ± 0.006	0.244 ± 0.006	6.693 ± 0.005	0.007 ± 0.005	2.197 ± 0.008	0.2 ± 0.005	1.624 ± 0.006	1.81 ± 0.007	81.77 ± 0.005	0.981 ± 0.006	0.49 ± 0.005	0.324 ± 0.006
B1	5.657 ± 0.005	0.229 ± 0.006	0.439 ± 0.005	0.003 ± 0.008	0.026 ± 0.006	0.529 ± 0.006	0.542 ± 0.005	1.98 ± 0.006	24.977 ± 0.006	10.66 ± 0.009	5.225 ± 0.008	0.025 ± 0.008
B2	1.136 ± 0.003	0.389 ± 0.007	0.447 ± 0.005	0.006 ± 0.009	0.039 ± 0.007	0.161 ± 0.006	0.372 ± 0.005	0.388 ± 0.008	103.20 ± 0.007	1.154 ± 0.006	0.12 ± 0.006	0.59 ± 0.005
B3	0.744 ± 0.009	0.044 ± 0.007	0.697 ± 0.005	0.045 ± 0.009	0.917 ± 0.006	0.254 ± 0.004	0.049 ± 0.006	2.258 ± 0.006	409.94 ± 0.006	0.851 ± 0.006	0.651 ± 0.006	0.904 ± 0.006
B4	2.29 ± 0.006	0.087 ± 0.006	0.304 ± 0.007	0.013 ± 0.008	0.039 ± 0.007	0.083 ± 0.009	0.015 ± 0.007	2.688 ± 0.004	22.48 ± 0.007	0.542 ± 0.009	2.351 ± 0.006	1.002 ± 0.004
C	3.419 ± 0.008	0.215 ± 0.008	2.808 ± 0.006	0.276 ± 0.006	2.22 ± 0.006	0.135 ± 0.008	0.162 ± 0.005	0.738 ± 0.009	113.47 ± 0.009	1.174 ± 0.006	2.551 ± 0.004	0.293 ± 0.008
Mean	2.160	0.198	1.673	0.044	0.833	0.203	0.338	1.150	101.945	1.942	1.712	0.479
ECR	200	–	100	–	2000	–	500	3000	2000	1000	–	10
WHO	10	30	10	–	3	–	50	2000	400	70	–	6
USEPA	–	–	15	–	5	–	100	1300	1600	–	–	–

SD = Standard deviation.

ECR = Environmental conservation rules, 2023, Bangladesh.

WHO = World Health Organization drinking water limit, 2023.

USEPA = United States Environment Protection Agency, 2018.

### Water pollution indices

**Fig 3:**
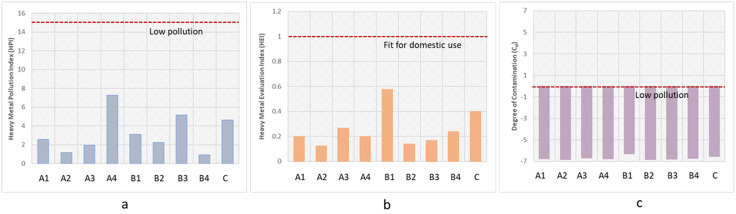
Water quality indices of nine sampling sites a. Heavy metal pollution index (HPI), b. Heavy metal evaluation index (HEI) and c. Degree of contamination (C_d_).

For assessment of the risks associated with inorganic pollution, WQI, HPI, and HEI are the most widely used parameters. A thorough assessment of PTEs and contamination trends in surface water ecosystems is made possible by these indices, which provide significant insights into the general environmental health and quality of aquatic habitats. In order to protect freshwater resources, their use facilitates the identification of possible pollution sources, the assessment of contamination levels, and the development of efficient management plans [33]. The water pollution level has been measured by using water quality indices (HPI, HEI, and C_d_) at the sampling contaminated sites, which are represented in Fig 3.

The Heavy Metal Pollution Index (HPI) is a crucial metric for evaluating how heavy metals collectively affect the overall quality of the water [34]. The HPI was calculated to assess the surface water’s overall quality concerning the presence of trace elements. The index is highly useful for tracking trends in metallic water contamination and quantifying them [34]. According to Zakir *et al*. (2020), HPI < 15 of any site means that the sampling site has low water contamination in terms of TM [33]. If HPI ranges from 15 to 30, then it means medium water contamination. HPI > 30 means very high contamination [33]. HPI of all the studied sampling sites is below 10, which indicates the water of the sampling sites is less contaminated.

The HEI was also analyzed to evaluate the overall quality of any surface or groundwater in the context of TM levels, which is highly beneficial to identifying and quantifying trends in water contamination [34]. The heavy metal evaluation index is used for the straightforward interpretation of the pollution index and level of pollution [24]. In terms of contamination level, HEI < 10 indicates low contamination, 10 < HEI < 20 indicates medium contamination, and 20 < HEI indicates significant contamination [33]. The threshold value was set at 1.0 to evaluate whether the surface and groundwater is fit or unfit for domestic use. HEI values below 1.0 are certified as “fit,” and those above 1.0 are labeled as “unfit” for domestic use [33]. Since the studied water samples have an HEI value below 1.0, it means the water of all sites is in a low contaminated state and still suitable for domestic usage in terms of TM contamination.

The degree of contamination is another parameter to measure the water contamination level. HPI, HEI, and C_d_ are closely related to each other to evaluate the water pollution in terms of heavy metals individually or integrated [24]. In the present study, all the C_d_ values are less than 1 means all the sites are of low water pollution, since the negative value of C_d_ indicates less water pollution [24].

The results of HEI, HPI, and C_d_ here denote the worthwhile notion of the PMIs in Bangladesh. Therefore, water is not being polluted so much by pharmaceutical effluents compared to other industries like textiles, dyeing, shipwrecking, tanneries, and so on.

### Human health risk assessment with water

River water, as well as water from their surrounding water bodies, is utilized by local people for a variety of things, including drinking, as raw water for industry, and other uses. Through physicochemical and biological processes, using polluted water can have adverse acute and chronic impacts on human health [35].

To determine exposure, the propensity of the hazardous components to coagulate in the human body, and the likelihood of both non-carcinogenic and carcinogenic risk, risk assessment was applied to the concentration of the examined element. The trace element concentration from the pharmaceutical outfalls’ surface water was used to determine the negative impact on human exposure *via* the three separate pathways—ingestion, inhalation, and skin exposure. The non-carcinogenic and carcinogenic risks were estimated through the different pathways for adult and children’s communities. The hazard quotient (HQ) for each pathway is displayed in S5 Table.The results revealed that As is the highest-absorbing material through the dermal route, and Mn is the highest-consuming material through the ingestion and inhalation route. However, HQ values for all pathways were within the acceptable limit (HQ < 1).

**Fig 4:**
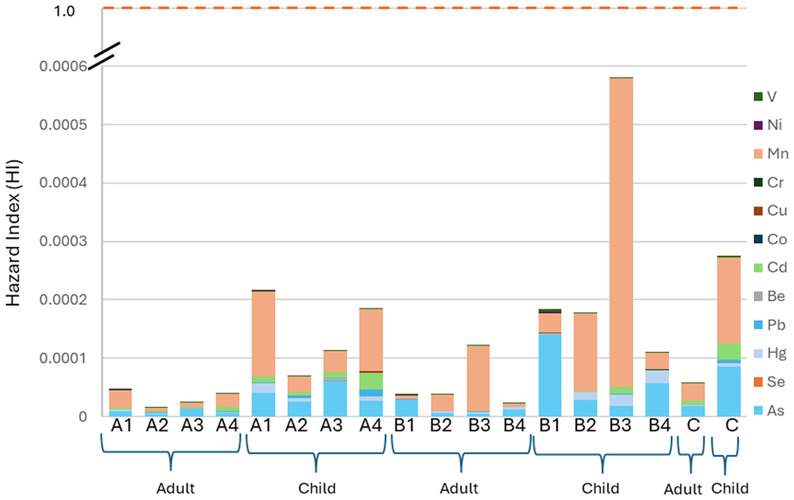
Hazard Index (HI) in the water collected from the sampling sites.

Fig 4 represents the Hazard Index (HI) or non-carcinogenic risk for adults and children in the respective area. The HI value for both adults and children maintained the following order: Mn> As> Cd > Hg > Pb > V > Cr > Ni > Se > Cu > Be > Co. The maximum HI value was found for Mn in the B3 sampling sites for both groups. Additionally, the HI values for the children are higher than those for the adults, likewise, previous studies, although a few studies were done with these twelve numbers elements [36,37]. This is because, concerning their body weight, children’s consumption limits are larger than those for adults, and they have a higher exposure rate [38].

The current study ensured that children were more susceptible than adults in terms of non-carcinogenic risk and that there might be very little probability of contamination through the three selected exposure paths in terms of a health hazard to those consuming river water.

**Fig 5:**
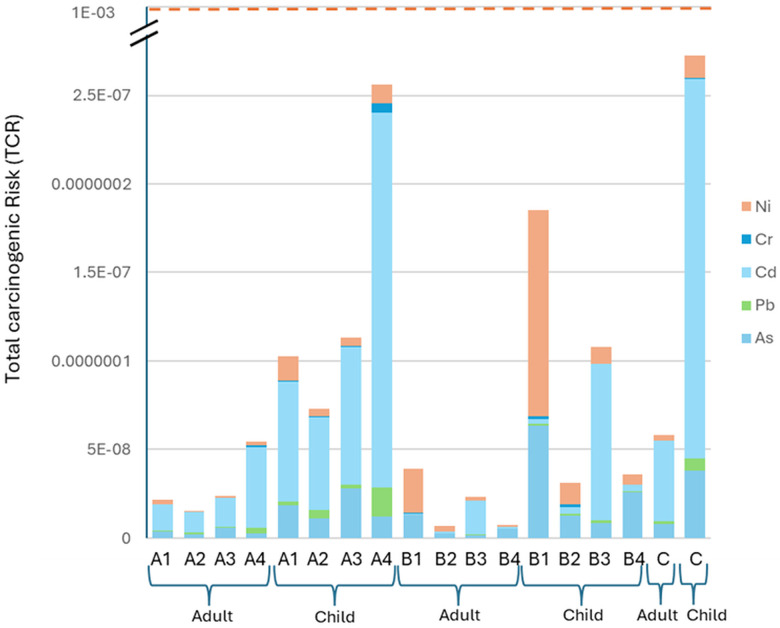
Total Carcinogenic Risk (TCR) in the water collected from the sampling sites.

The total CR was estimated for only As, Pb, Cd, Cr, and Ni since the carcinogenic slope factor of only these elements was available. The risk evolves from lifetime exposure to a metal with a specific cancer slope factor [37]. Fig 5 illustrates the total carcinogenic risk of the inhabitants of the mentioned sampling areas occurring from TMs. The total CR values ranged from 2.32 × 10^−10^ to 1.72 × 10^−8^ for adults and 1.09 × 10^−9^ to 8.04 × 10^−8^ for children, while the maximum CR value was found in the B1 water sample for of child group. This carcinogenic risk assessment for any individual carcinogen determines the concern of developing cancer. According to USEPA, a 10^−6^ to 10^−4^ lifetime carcinogenic risk is acceptable, and a carcinogenic risk greater than 10^−4^ defines the potential probability of carcinogenic risk [39].

**Fig 6:**
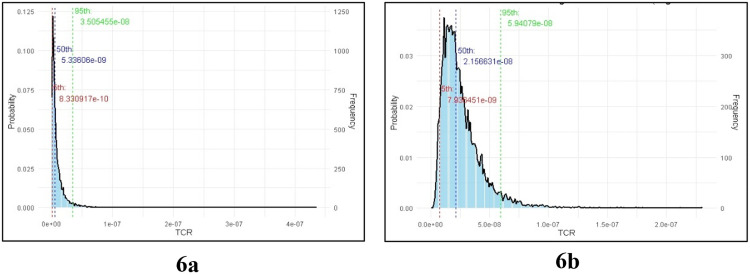
Monte Carlo Simulation for TCR on a. Adults, b. Child.

The total cancer risk (TCR) for adults exposed to trace metals in water was estimated using a Monte Carlo simulation with a log-normal distribution, which was represented in Fig 6a Based on the mean (1.03354 × 10^−8^) and standard deviation (1.68502 × 10^−8^) of the measured TCR values, the simulation produced 10,000 iterations. 8.33 × 10^−10^, 5.33 × 10^−9^, and 3.51 × 10^−8^ were found to represent the 5^th^, 50^th^, and 95^th^ percentiles of the simulated TCR, respectively. The frequency of simulated values on the secondary y-axis and the probability density of TCR on the primary y-axis are depicted in the simulation results histogram. Fifty people (about 0.5%) out of 10,000 simulations exceeded the cancer risk threshold of 1 × 10^−6^ suggesting that a small portion of the population may be at heightened risk for cancer based on this exposure pathway.

The total cancer risk (TCR) for children exposed to trace metals in water was calculated using a Monte Carlo simulation. 10,000 iterations of the simulation were generated using the mean (2.62018 × 10^−8^) and standard deviation (1.76964 × 10^−8^) of the measured TCR values for children. Children’s simulated TCR values fell into the 5^th^, 50^th^, and 95^th^ percentiles at 7.94 × 10^−9^, 2.12 × 10^−8^, and 5.94 × 10^−8^, respectively. The simulation results’ histogram (Fig 6b) sheds light on the frequency and probability distribution of TCR values.

A tiny percentage of the population may be at higher risk for cancer as a result of trace metal contamination in water, as indicated by the simulation, which showed that about 30 out of 10,000 children (or 0.3%), above the cancer risk threshold of 1 × 10^−6^. These findings emphasize how crucial it is to consider exposure hazards to children, a potentially more sensitive group. For the small percentage of kids who might be at higher risk of developing cancer due to these environmental variables, exposure should be decreased by ongoing monitoring and mitigation techniques.

The children group in the studied area is more inclined to be non-carcinogenic than the adult group, which exactly matches a previous health risk assessment study by Sharma *et al*. (2019) [40]. Although the adults are more inclined to be at carcinogenic risk according to the Monte Carlo simulation, the risks are still below the threshold value. However, since the HQ, HI, and CR may eventually outweigh the risks due to rapid industrialization and climate change, children should be kept as far away from industrially contaminated surface water as possible.

### Trace element concentration in sediment samples

The average concentration of 12 trace elements was determined in the sediment samples collected from the nine sampling sites. Table 2 shows the concentration of the trace elements in the sediments of the sampling sites as follows: Se (0.04–0.23 mg/kg), Be (0.05–0.79 mg/kg), Cu (1.39–38.89 mg/kg), Mn (53.58–200.47 mg/kg), As (0.08–3.34 mg/kg), Pb (1.11–78.35 mg/kg), Cd (0.09–1.36 mg/kg), Co (1.22–9.39 mg/kg), Cr (0.26–20.53 mg/kg), Ni (0.2–75.68), V (0.8–24.42 mg/kg), Hg (0.08–0.96 mg/kg). The average concentration of trace elements in the sampling sites showed the following descending trend: Mn > Ni > Cu > Pb > V > Cr > Co> As> Cd > Hg > Be > Se.

**Table 2:** Trace element concentration (Mean ± SD) in the sediment samples (in mg/kg).

Sampling sites	Se	Be	Cu	Mn	As	Pb	Cd	Co	Cr	Ni	V	Hg
A1	0.23 ± 0.001	0.79 ± 0.002	1.39 ± 0.001	53.58 ± 0.005	3.34 ± 0.002	21.9 ± 0.001	0.16 ± 0.002	3.71 ± 0.008	2.84 ± 0.006	10.2 ± 0.007	2.86 ± 0.005	0.19 ± 0.004
A2	0.15 ± 0.005	0.3 ± 0.007	20.75 ± 0.002	120.35 ± 0.004	2 ± 0.009	78.35 ± 0.003	1.36 ± 0.007	4.68 ± 0.004	12.28 ± 0.006	75.68 ± 0.009	24.42 ± 0.005	0.8 ± 0.002
A3	0.04 ± 0.006	0.12 ± 0.002	8.77 ± 0.002	78.08 ± 0.009	0.51 ± 0.008	1.11 ± 0.005	0.84 ± 0.006	2.76 ± 0.002	3.99 ± 0.001	0.2 ± 0.001	7.22 ± 0.002	0.06 ± 0.003
A4	0.22 ± 0.001	0.31 ± 0.004	38.89 ± 0.001	192.2 ± 0.006	1.4 ± 0.007	6.51 ± 0.002	0.15 ± 0.003	6.42 ± 0.006	3.09 ± 0.003	29.28 ± 0.003	6.17 ± 0.005	0.57 ± 0.001
B1	0.19 ± 0.001	0.42 ± 0.001	38.6 ± 0.005	187.4 ± 0.007	3.16 ± 0.002	19.38 ± 0.004	0.09 ± 0.002	8.84 ± 0.003	16.67 ± 0.001	19.73 ± 0.005	21.74 ± 0.007	0.08 ± 0.004
B2	0.09 ± 0.002	0.18 ± 0.004	38.29 ± 0.005	200.47 ± 0.003	0.08 ± 0.002	7.11 ± 0.003	1.05 ± 0.003	5.39 ± 0.001	20.53 ± 0.001	15.54 ± 0.003	2.78 ± 0.003	0.29 ± 0.001
B3	0.06 ± 0.002	0.14 ± 0.002	3.89 ± 0.002	88.86 ± 0.002	0.27 ± 0.001	1.2 ± 0.002	0.96 ± 0.002	9.39 ± 0.001	8.32 ± 0.003	12.17 ± 0.001	0.8 ± 0.003	0.96 ± 0.002
B4	0.05 ± 0.001	0.05 ± 0.001	13.2 ± 0.005	75.61 ± 0.002	0.11 ± 0.001	1.32 ± 0.001	0.32 ± 0.005	2.72 ± 0.002	0.26 ± 0.002	12.4 ± 0.002	13.73 ± 0.005	0.25 ± 0.005
C	0.04 ± 0.004	0.12 ± 0.005	4.96 ± 0.002	64.29 ± 0.001	0.51 ± 0.002	1.11 ± 0.002	0.84 ± 0.002	1.22 ± 0.003	1.63 ± 0.001	0.2 ± 0.001	10.09 ± 0.003	0.13 ± 0.003
ASV^a^	0.6	3	45	850	13	20	0.03	19	90	68	130	0.4
TRV^b^	–	–	28	–	8.2	21	1	–	8.1	20.9	–	0.15
TEL^c^	–	–	35.7	–	5.9	35	0.6	–	37.3	18	–	0.17

SD = Standard deviation.

a. ASV = Average Shale Value, b. TRV = Toxicity Reference Value proposed by USEPA, c. TEL = Threshold Effect Level proposed by USEPA.

In this table, the concentrations were also compared with the average shale value (ASV) of elements in the Earth’s crust mentioned in Turekian and Wedepohl (1961) [41], toxicological reference value (TRV), and threshold effect level (TEL) proposed by USEPA [42,43]. The concentrations of the trace elements are below ASV except for Cd, and the concentrations of Hg exceeded the TRV. In addition, Cd, Ni, and Hg contents are higher than the TEL values. The mean concentration in the studied area varied from other studies conducted in the various rivers of Bangladesh. The mean concentration in the investigated area varied from other research conducted in Bangladesh’s various rivers. Industrial effluents from battery, electroplating, ferrous and non-ferrous manufacturing units, and pigments increase the Cd and Hg content [9]; small-scale industries like metal processing may take part in Ni contamination [10].

To distinguish between non-polluted, moderately polluted, and heavily polluted sediments, the USEPA Toxicology suggested the sediment quality classification recommendations for the different elements, including Pb, Cu, As, Cd, Ni, and Mn concentration [9] which are illustrated in Table 3. Comparing this table with the value of Table 3, it can be seen that sampling site A2 is heavily contaminated with Pb and Ni. Similarly, A4, B1, and B2 are moderately polluted with Cu, whereas the other sampling sites are non-polluted. Moderate, As pollution was found in A1 and B1. Overwhelmingly, all the sampling sites are considered non-polluted with Mn and Cd according to the USEPA classification.

**Table 3:** Classification of sediment quality guidelines according to USEPA toxicity.

USEPA toxicity classifications			
Element	Non-polluted	Moderately polluted	Heavily polluted
Pb	< 40	40-60	>60
Cu	< 25	25-50	>50
As	< 3	3-8	>8
Cd	–	–	>6
Ni	< 20	20-50	>50
Mn	< 300	300-500	>500

**Fig 7:**
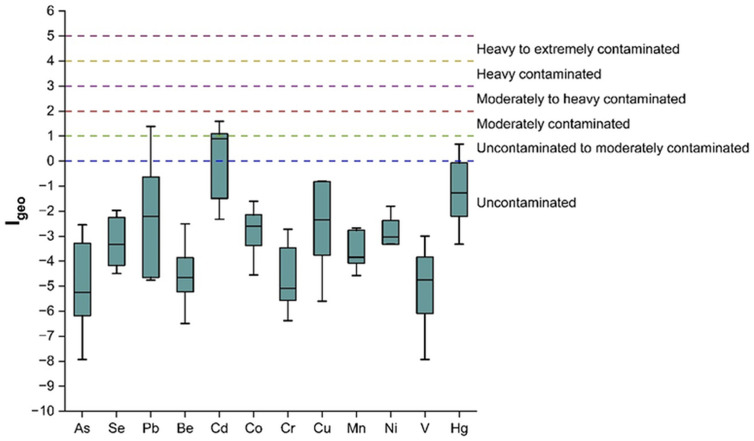
Box-whisker plot of Geo-accumulation index (I_geo_) of the trace elements in the sampling sites.

Geo-accumulation index (I_geo_) has been used to evaluate the quality of sediment; however, since the calculation of I_geo_ involves a log function and a background multiplication of 1.5, it is not easily comparable to the other metal enrichment indices [44]. A Box-Whisker plot (Fig 7) has been illustrated, showing the average I_geo_ in the nine sampling sites for each TM. The Fig indicates that the I_geo_ value of Cd was the highest (moderately contaminated) followed by Hg and Pb. Agricultural activities, industrial effluents, metallurgical processes, and household wastes may be the potential sources of higher I_geo_ values for Pb and Cd [45]. Similarly, Fu *et al* identified Pb and Cd as the most responsible for the ecological risk of their study area in China [46]. Since Cd pollution distribution is widespread, and the potential ecological risk due to Cd pollution poses an extensive threat to the environment, it is important to identify the contributing factors and the amount of their impact on soil-Cd contamination to reduce the level of soil-heavy metals from steadily rising. Moreover, I_geo_ is significantly impacted by human engineering efforts, which frequently lead to moderate to heavy contamination [47].

**Fig 8:**
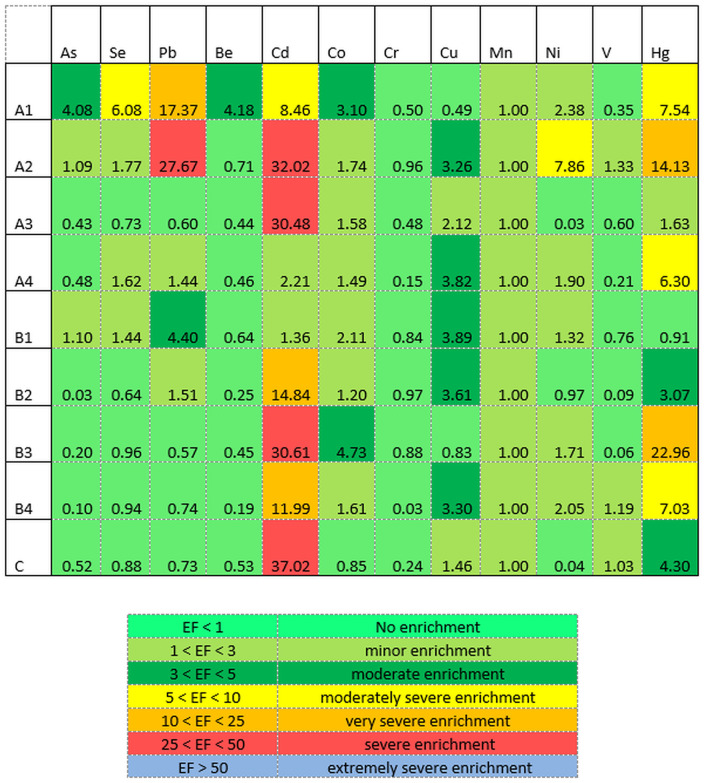
Heat map of enrichment factor (EF) values of the nine sampling sites.

The normalized enrichment factor is applied to distinguish between element sources coming from anthropogenic and natural sources. This entails normalizing the sediment to reference elements including Al, Fe, Mn, Ti, Sc, Li or Cs [44]. In this study, Mn was considered as a reference element to calculate anthropogenic metal enrichment according to Salati and Moore (2010 [48]. A heat map showing the EF values has been revealed in Fig 8. EF values of Se, Be, As, Cr, and V (except in A1, A2, A4, B1 for Se; A1 for Be; A1, A2, B1 for As; A2, B4, C for V) are less than 1, suggesting no enrichment of these elements. EF values of Se at A2 (1.77), A4 (1.62), B1 (1.44); Cu at A3 (2.12) and C (1.46); Mn at all sites showed minor enrichment as EF is greater than 1 but less than 3. Minor enrichment occurred also for As at A2, B1; Pb at A4, B2; Cd at A4 only; Co at all sites except A1, B3, and C; Ni at A1, A4, B1, B3; V at A2, B4, C and Hg at A4. The EF values of Be at A1; Cu at A2, A4, B1, B2, B4; As at A1, Cd at A4, Co at A1, B3, and Hg at B2, C exhibited moderate enrichment since it lie between 3 and 5. Sampling site A1 was moderately severely enriched with Se (6.08), Cd (8.46), and Hg (7.53), similar to A2 with Ni (7.86); A4 with Hg (6.3); B4 with Hg (7.03). EF value of Cd at B2, B4; Pb at A1 and Hg at A2, B3 indicated severe enrichment (10 < EF < 25) where sampling site A2 was very severely enriched with Pb and Cd (27.67 and 32.02, respectively) just as like A3, B3 and C with Cd (30.48, 30.61 and 37.02). The main sources of these elements are municipal and household garbage, burning fossil fuels, and runoff from agriculture [44], and pharmaceutical effluents [49] Although most PMIs strictly follow the effluent treatment guidelines [15]. Based on the EF values, the majority of sampling sites fall within the no enrichment to minor enrichment areas. In this study, no other elements were enriched like Cd, similar to previous studies [8,36,45]. EF > 1.5 indicates that the sampling sites are contaminated with the respective elements from anthropogenic roots [8]. Several factors can be responsible for the high concentration of Cd since the geological condition, topography, human engineering activities, and river transport system affect the distribution of Cd [44].

**Fig 9:**
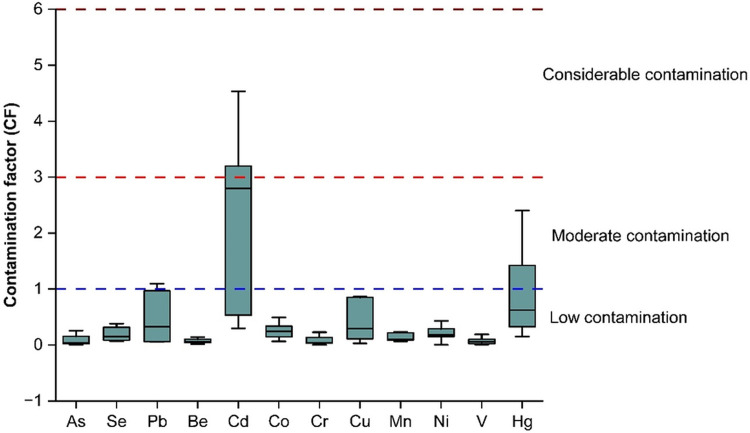
Box-whisker plot of contamination factor (CF) of the trace elements in the sampling sites.

A Box-Whisker plot (Fig 9) has been demonstrated to show the average contamination factor in the nine sampling sites of individual trace elements. Similar to the I_geo_ values, CF values of Cd were the most prominent, followed by Hg and Pb, Cu. Co, Se, Mn, and Ni showed almost similar contamination, whereas As, Be, Cr, and V contamination were minimal. However, the soil contamination according to CF values was analogous to that of I_geo_ values.

**Fig 10:**
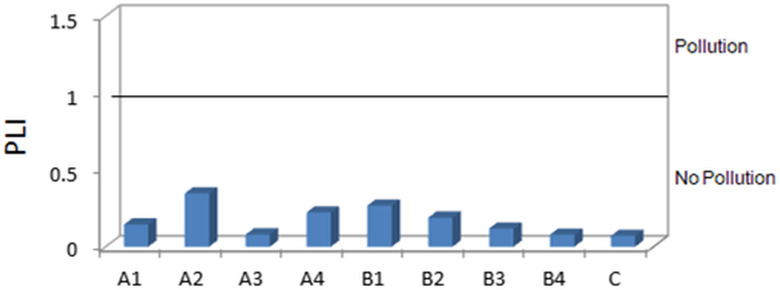
Pollution Load Index (PLI) at the sampling sites.

The pollution load index (PLI) determines the integrated pollution by elements of any sampling sites, which are shown in Fig 10. The graph revealed that the sediments from all the sampling sites were uncontaminated (PLI < 1) since the PLI values ranged from 0.07 to 0.34. The maximum PLI was at A2, which was derived from the higher CF value for Pb and Cd. The PLI can signify an overall illustration of the sediment quality that affects the residents [45]. Therefore, although the PLI value indicates no pollution at present, the individual pollution of Cd and Pb can be a matter of concern shortly.

**Fig 11:**
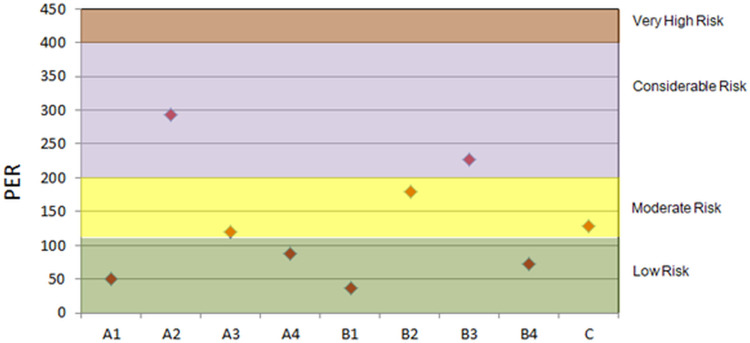
Potential Ecological Risk (PER) at the sampling sites.

The potential ecological risk, illustrated in Fig 11, showed the values ranged from 35.61 to 293.18. The highest PER was at sampling site A2, followed by B3, suggesting considerable ecological risk (200 ≤ PER < 400) in these areas. Sampling sites A3 (121.29), B2 (178.85), and C (127.33) were at moderate risk. Okoro et al. reported similar results at several sampling points in Kwara State, Nigeria [45]. The PER values of less than 110 of the remaining areas indicate low ecological risk. The degree of pollution in terms of potential ecological risk followed a decreasing order of A2 > B3 > B2 > C > A3 > A4 > B4 > A1 > B1. Even though none of the analyzed pharmaceutical outfall regions posed a particularly high ecological danger, ongoing observation of these locations will be beneficial for the ecosystem’s preservation. The sample sites A2 and B3 should also be taken into account by the appropriate authorities for routine PER evaluation to prevent PER from these areas from entering the zone of extremely high risk. However, it is well known that, EF or I_geo_ demonstrate the enrichment of specific element in a specific zone. So, t is quite possible for an element to get highly enriched but still within the tolerable limit. For this reason, PER values of some site indicates low ecological risk even though the enrichment of element is considerably high.

### Statistical results and source identification

The correlation matrix was performed to evaluate the relationships between the elements as well as to identify their source of origin and distribution pattern [37,50]. Pearson’s correlation matrix among the trace elements in water and sediment was represented in Tables 4 and 5, respectively. In water, a strong positive correlation (p < 0.01) was detected with As-Co (r = 0.553), As-Ni (r = 0.831), and As-V (0.928), as well as Pb-Cd (r = 0.819), Pb-Cr (r = 0.831), Be-Cd (0.607), Co-Ni (r = 0.861), Co-V (r = 0.616), Mn-Hg (r = 0.496), and Ni-V (r = 0.841), suggesting the same anthropogenic sources of the elements [37]. A negative correlation occurred in the As-Mn (r = −0.4), As-Hg (r = −0.541), Se-Cu (r = 0.489) and Ni-Hg (r = −0.468) pairs [50].

**Table 4:** Pearson’s correlation matrix among the trace element content in the water.

Correlations												
	As	Se	Pb	Be	Cd	Co	Cr	Cu	Mn	Ni	V	Hg
As	1											
Se	.039	1										
Pb	−.235	.167	1									
Be	.236	−.028	.174	1								
Cd	−.147	−.015	.819^**^	.607^**^	1							
Co	.553^**^	.210	−.124	−.191	−.203	1						
Cr	−.033	.378	.831^**^	−.194	.477^*^	.239	1					
Cu	.195	−.489^**^	.048	−.137	−.060	.286	.232	1				
Mn	−.400^*^	−.329	−.076	.139	.194	.141	−.135	.281	1			
Ni	.831^**^	.164	−.206	−.131	−.322	.861^**^	.177	.292	−.205	1		
V	.928^**^	−.094	−.266	.163	−.243	.616^**^	−.105	.306	−.361	.841^**^	1	
Hg	−.541^**^	−.261	−.279	−.147	−.231	−.184	−.321	.367	.496^**^	−.468^*^	−.370	1

**. Correlation is significant at the 0.01 level (2-tailed).

*. Correlation is significant at the 0.05 level (2-tailed).

**Table 5:** Pearson correlation matrix among the trace element content in the sediment.

Correlations												
	As	Se	Pb	Be	Cd	Co	Cr	Cu	Mn	Ni	V	Hg
As	1											
Se	.851^**^	1										
Pb	.497^**^	.409^*^	1									
Be	.898^**^	.851^**^	.344	1								
Cd	−.480^*^	−.536^**^	.378	−.467^*^	1							
Co	.239	.348	.048	.146	−.127	1						
Cr	.131	.142	.321	.047	.327	.549^**^	1					
Cu	.132	.408^*^	.120	−.012	−.182	.438^*^	.633^**^	1				
Mn	.098	.393^*^	.085	−.007	−.103	.561^**^	.707^**^	.977^**^	1			
Ni	.309	.403^*^	.914^**^	.144	.357	.210	.337	.344	.317	1		
Zn	−.025	.090	.567^**^	−.278	.348	.098	.519^**^	.700^**^	.632^**^	.722^**^		
V	.378	.155	.664^**^	−.013	.051	−.008	.220	.288	.160	.622^**^	1	
Na	.437^*^	.517^**^	.106	.468^*^	−.194	.437^*^	.793^**^	.672^**^	.715^**^	.070	.008	
K	.386^*^	.701^**^	−.065	.400^*^	−.449^*^	.598^**^	.097	.502^**^	.576^**^	.125	−.209	
Hg	−.153	.057	.377	−.127	.442^*^	.505^**^	.115	−.025	.089	.582^**^	−.053	1

**. Correlation is significant at the 0.01 level (2-tailed).

*. Correlation is significant at the 0.05 level (2-tailed).

In the sediment samples, as had a significant positive correlation (p < 0.01) with Se, Pb, and Be (r = 0.851, 0.497, and 0.898, respectively). Also, Se with Be (r = 0.851); Pb with Ni and V (r = 0.914 and 0.664 respectively); Co with Cr, Mn and Hg (r = 0.549, 0.561 and 0.505 respectively); Cr with Cu and Mn (r = 0.633 and 0.707); Cu with Mn (r = 0.977) and Ni with V and Hg (r = 0.622 and 0.582 respectively) showed a strong positive correlation. An opposite significant correlation was exhibited in Se and Cd (r = −0.530) only. The relevant sources, individual characteristics, and mutual dependency on transportation through the water bodies may also contribute to a similar level of contamination from the two particular metals [51].

**Fig 12:**
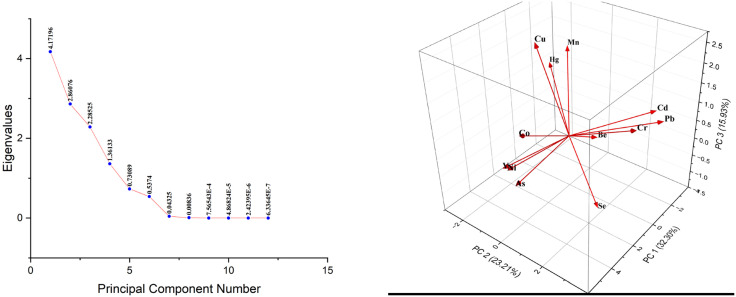
Scree plot and PCA loading plot of trace elements in water samples.

**Fig 13:**
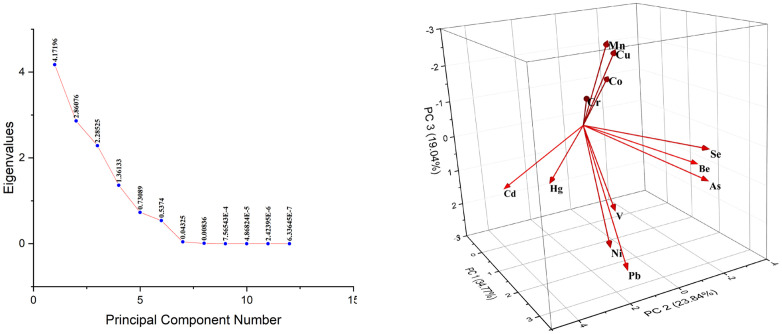
Scree plot and PCA loading plot of trace elements in sediment samples.

**Fig 14:**
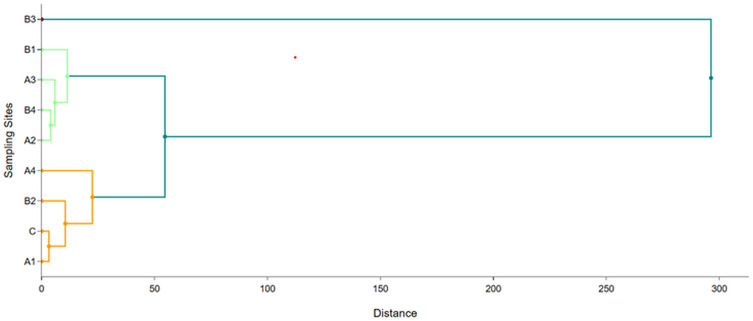
A hierarchical cluster (Dendrogram) among the sampling sites for water.

**Fig 15:**
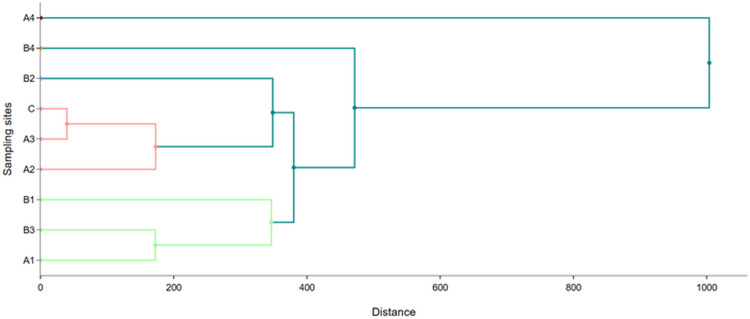
A hierarchical cluster (Dendrogram) among the sampling sites for sediment.

Principal component analysis (PCA) and Cluster analysis (CA) are multivariate statistical analyses that have been accomplished to contribute to expressive statistics concerning trace element pathways and their sources [52]. PCA is carried out using the eigenvalues of the correlation matrix, which establishes how the variables in a multidimensional dataset are correlated with one another [53]. From the scree plot, the number of principal components can be identified. The PCA results of the elements with factors were revealed in S6 Table, and Figs 14 and 15 illustrate the scree plot and PCA loading plot of the water and sediment samples, respectively. S7 Table and Fig 12 show the results of PCA analysis, in water samples, PC 1 contributed to 32.30% of the total variance, mainly with loadings of Ni, V, and As (0.490, 0.468, and 0.467, respectively). PC 2 took part in 23.21% of the total variance with high loadings of Pb, Cr, and Cd (0.526, 0.473, and 0.453, respectively), and PC 3 contributed 15.93% of the total variance with maximum loadings of Cu (0.612) and Mn (0.445). The contribution of PC 1, PC 2, and PC 3 in sediment samples was explained in S7 Table and Fig 13. PC 1 imparted 34.77% whereas PC 2 and PC 3 explained 23.84% and 19.04% of the total variance, respectively, in sediment samples. Se, Ni, and As dominated PC 1 with loadings of 0.379, 0.355, and 0.339, correspondingly, where Cd, Hg, and Cr controlled PC 2 with loadings of 0.488, 0.337, and 0.260, respectively; Pb, Ni, and V controlled PC 3 with loadings of 0.444, 0.315, and 0.248, respectively. The results explained the internal relationships between the metals and said that additional geogenic processes as well as untreated industrial and municipal wastes could be the origins of the elements. Hence, anthropogenic activities and industrial and agricultural chemicals can both be attributed to the element’s formation [37].

Hierarchical cluster analysis (HCA), a well-known statistical approach, helps in identifying groupings of samples that behave or exhibit similar traits, and the structural properties of the samples or variables are subsequently quantified [54]. HCA was applied in this study to identify the similarity between the sampling sites using the Ward linkage method of Euclidean square root distance. Figs 14 and 15 illustrate the dendrograms evolved from cluster analysis of the sampling sites for water and sediment, respectively. In the case of the water, three clusters were found where each group showed similarity in contamination sources like cluster 1 (A1, C, B2, and A4), cluster 2 (A2, B4, A3, and B1), and cluster 3 (B3). Since sampling site C is the outfall where several individual USFDA-approved pharmaceuticals are integrated throw their effluents, its similarity with A1 and A4 is reasonable. Interestingly, USFDA-not-approved pharmaceutical B2 showed a linkage with A1 and A4 (USFDA-approved), and B1 and B4 had a connection with A2 and A3. B3 had the most uncommon approach, showing dissimilarity with any other sites.

In the case of sediment, the sampling sites were grouped into five clusters where A1, B3, and B1 were in Cluster 1; A2, A3, and C were in Cluster 2, and B2, B4, and A4 were in Clusters 3, 4, and 5, respectively. The findings showed that the studied pharmaceuticals were categorized differently in terms of releasing trace elements, despite being classified according to the USFDA. Since all the sampling sites are located in the acute freshwater region, some common anthropogenic activities like throwing industrial waste, sand accumulation, and nearby different types of industries would stress the increase metal concentrations [37].

## Conclusion

Finally, the evaluation of the ecological impact, health risk, and trace element concentration yields the following important conclusions: 1. Physicochemical parameters along with water quality indices demonstrated low levels of pollution of the water samples collected from chosen sites. Even though the health risk to both adults and children by utilizing this water is below the boundary line, still all are facing some sort of risk, and children are at higher risk than adults. 2. The study of the quality of sediment samples demonstrated that the examined PTEs pose little ecological danger to the area under study. PTEs are still within the acceptable range, while some of them display substantial enrichment (high I_geo_ and EF values). 3. According to principal component analysis, Ni, V, and As are the main contributors of current PTE pollution in water, while Se, Ni, and As dominated other PTEs in terms of PTE pollution in sediment. 4. Hierarchical cluster analysis visualized the similarities of sampling sites in terms of water and sediment based on their PTE profile.

Based on the conclusions mentioned above, one can easily claim that the PMIs in Bangladesh presently comply with waste management regulations, reducing direct environmental damage. Ongoing oversight and management are, nevertheless, required to avoid extended exposure and potential hazards in the future. To stop additional soil pollution and the ensuing deterioration of the ecosystem, efforts should be directed toward lowering the discharge of lead (Pb) and cadmium (Cd). A thorough classification system that prioritizes efficient waste management techniques in addition to product quality is required for pharmaceuticals. This all-encompassing strategy can lessen the PMI’s environmental impact. This study highlights the significance of more research, even though it sheds light on the detrimental effects of pollution caused by the pharmaceutical business. There are some limitations to this research also. The pharmaceutical sector is the primary source of trace metal (TM) contamination in the research, with other potential contaminants, such as organic pollutants and other hazardous byproducts, going unexplored. Furthermore, the study’s focus is restricted to limited areas of Bangladesh, which might not accurately reflect the broader encyclopedic context. Besides, seasonal variation should be studied, as the pollution level of any specific contaminated area largely depends on it. Other limitations include a lack of longitudinal data and the potential for unaddressed cumulative exposure. However, subsequent research endeavors ought to focus on assessing the hazards linked to organic pollutants and, additionally, possibly detrimental consequences of pharmaceutical manufacturing. Understanding the cumulative and long-term environmental repercussions will be aided by undertaking longitudinal studies and broadening the research to include other regions. This wider focus will help to clarify the consequences of the pharmaceutical sector on the environment and provide information for more focused mitigation strategies. The findings highlight the need for stricter monitoring, regulatory enforcement, and targeted interventions to reduce public exposure to PTEs.

## Supporting information

S1 Table Sampling location of the current study. (PDF)

S2 Table LOD and LOQ values of analyzed trace metals by ICP-MS. (PDF)

S3 Table Heavy metal concentration (mean ± standard deviation) in certified reference material (NIST SRM-1640). (PDF)

S4 Table Heavy metal concentration (mean ± standard deviation) in certified reference material (GBW07309). (PDF)

S5 Table Physicochemical Parameters (Mean ± SD) of water samples. (PDF)

S6 Table Hazard Quotient of adults and children for different exposure pathways. (PDF)

S7 Table PCA of two components with the respective loading of metals of the study area. (PDF)

S1 File Supporting information about the method. (PDF)
