# Supplementary material for: Investigative health and ecological risk assessment of trace elements in pharmaceutical deposition near Dhaka: An endemic industrial surge of Bangladesh
Source: PLoS One. 2026 Jan 5;21(1):e0338816. doi: 10.1371/journal.pone.0338816 (PMC12768289; doi:10.1371/journal.pone.0338816)
Supplement: S1 Table — (PDF) [file pone.0338816.s001.pdf]

S1 Table: Sampling location of the current study

Sample site	Location Name	Latitude	Longitude	Distance from river
A1	Tongi, Gazipur	23.90255°	90.39040°	5.28 km from Turag
A2	Tongi, Gazipur	23.90126°	90.38938°	5.14 km from Turag
A3	Godnail, Narayanganj	23.63807°	90.51710°	1.12 km from Shitalakhya
A4	Siddhirganj, Narayanganj	23.66961°	90.52780°	444.65 from Shitalakhya
B1	Tongi, Gazipur	23.89915°	90.39905°	6.32 km from Turag
B2	Narayanganj Sadar	23.62437°	90.50748°	87.24 m from Shitalakhya
B3	Rupganj, Narayanganj	23.73354°	90.51037°	871.48 m from Shitalakhya
B4	Rupganj, Narayanganj	23.74377°	90.51436°	151.81 from Shitalakhya
C	Tongi, Gazipur	23.90213°	90.39419°	6.28 km from Turag river
