# Supplementary material for: Investigative health and ecological risk assessment of trace elements in pharmaceutical deposition near Dhaka: An endemic industrial surge of Bangladesh
Source: PLoS One. 2026 Jan 5;21(1):e0338816. doi: 10.1371/journal.pone.0338816 (PMC12768289; doi:10.1371/journal.pone.0338816)
Supplement: S2 Table — (PDF) [file pone.0338816.s002.pdf]

S2 Table: LOD and LOQ values of analyzed trace metals by ICP-MS

Element	LOD (µg/L)	LOQ (µg/L)
**As**	0.08	0.30
**Pb**	0.0035	0.013
**Cd**	0.005	0.04
**Cr**	0.005	0.019
**Ni**	0.005	0.041
**Co**	0.004	0.011
**Cu**	0.08	0.24
**Mn**	0.021	0.051
**Hg**	0.005	0.015
**Se**	0.005	0.019
**Be**	0.001	0.002
**V**	0.008	0.014
