# Supplementary material for: Investigative health and ecological risk assessment of trace elements in pharmaceutical deposition near Dhaka: An endemic industrial surge of Bangladesh
Source: PLoS One. 2026 Jan 5;21(1):e0338816. doi: 10.1371/journal.pone.0338816 (PMC12768289; doi:10.1371/journal.pone.0338816)
Supplement: S3 Table — (PDF) [file pone.0338816.s003.pdf]

S3 Table: Heavy metal concentration (mean ± standard deviation) in certified reference material (NIST-SRM-1640)

Element	Certified value (µg/kg)	Measured value (µg/kg)	Mean recovery (%)
As	8.010 ± 0.067	8.17 ± 1.201	102
Pb	12.005 ± 0.040	12.245 ± 0.018	102
Cd	3.961 ± 0.072	4.00 ± 0.054	101
Cr	40.22 ± 0.28	41.024 ± 1.124	102
Ni	25.12 ± 0.12	24.618 ± 0.097	98
Co	20.08 ± 0.24	20.481 ± 0.065	102
Cu	85.07 ± 0.48	85.921 ± 1.414	101
Mn	40.07 ± 0.35	39.669 ± 0.011	99
Se	19.97 ± 0.16	19.371 ± 0.098	97
Be	3.002 ± 0.027	3.122 ± 0.059	104
V	14.93 ± 0.21	15.229 ± 1.023	102
