# Supplementary material for: Investigative health and ecological risk assessment of trace elements in pharmaceutical deposition near Dhaka: An endemic industrial surge of Bangladesh
Source: PLoS One. 2026 Jan 5;21(1):e0338816. doi: 10.1371/journal.pone.0338816 (PMC12768289; doi:10.1371/journal.pone.0338816)
Supplement: S4 Table — (PDF) [file pone.0338816.s004.pdf]

S6 Table: Hazard Quotient of adults and children for different exposure pathways

Sampling sites		As	Se	Hg	Pb	Be	Cd	Co	Cr	Cu	Mn	Ni	V
A1	Ingestion												
	Adult	7.45E-06	7.47E-08	3.47E-06	3.65E-07	3.0E-09	1.93E-06	7.07E-09	6.34E-08	7.27E-09	3.11E-05	8.4E-08	2.95E-07
	Child	3.47E-05	3.49E-07	1.62E-05	1.70E-06	1.40E-08	9.01E-06	3.30E-08	2.96E-07	3.39E-08	1.45E-04	3.95E-07	1.37E-06
	Dermal												
	Adult	8.92E-07	2.98E-10	1.38E-08	1.46E-09	1.20E-11	7.70E-09	2.82E-11	2.53E-10	2.90E-11	1.24E-07	3.38E-10	1.17E-09
	Child	5.84E-06	1.95E-09	9.09E-08	9.56E-09	7.87E-11	5.04E-08	1.84E-10	1.65E-09	1.90E-10	8.13E-07	2.21E-09	7.71E-09
	Inhalation												
	Adult	3.84E-09	3.85E-11	1.79E-09	1.88E-10	1.55E-12	9.95E-10	3.64E-12	3.27E-11	3.74E-12	1.60E-08	4.36E-11	1.52E-10
	Child	1.79E-08	1.79E-10	8.36E-09	8.8E-10	7.24E-12	4.64E-09	1.70E-11	1.52E-10	1.74E-11	7.48E-08	2.03E-10	7.10E-10
**A2**	Ingestion												
	Adult	4.54E-06	4.71E-08	1.47E-06	7.47E-07	7.94E-09	1.49E-06	2.26E-09	2.51E-08	2.95E-09	5.62E-06	2.86E-08	2.85E-07
	Child	2.12E-05	2.19E-07	6.86E-06	3.49E-06	3.70E-08	6.98E-06	1.05E-08	1.17E-07	1.37E-08	2.62E-05	1.33E-07	1.33E-06
	Dermal												
	Adult	5.43E-07	1.88E-10	5.86E-09	2.98E-09	3.17E-11	5.96E-09	9.01E-12	1.00E-10	1.17E-11	2.24E-08	1.14E-10	1.13E-09
	Child	3.56E-06	1.23E-09	3.84E-08	1.95E-08	2.07E-10	3.90E-08	5.90E-11	6.56E-10	7.71E-11	1.47E-07	7.49E-10	7.45E-09
	Inhalation												

	Adult	2.34E-09	2.42E-11	7.57E-10	3.85E-10	4.09E-12	7.70E-10	1.16E-12	1.29E-11	1.52E-12	2.89E-09	1.47E-11	1.47E-10
	Child	1.09E-08	1.13E-10	3.53E-09	1.79E-09	1.91E-11	3.59E-09	5.43E-12	6.03E-11	7.10E-12	1.35E-08	6.90E-11	6.86E-10
**A3**	Ingestion												
	Adult	1.13E-05	3.53E-08	4.01E-07	3.23E-07	1.36E-09	2.22E-06	1.25E-09	3.78E-08	7.68E-09	7.54E-06	3.12E-08	2.05E-07
	Child	5.30E-05	1.64E-07	1.87E-06	1.50E-06	6.39E-09	1.03E-05	5.85E-09	1.76E-07	3.58E-08	3.52E-05	1.45E-07	9.58E-07
	Dermal												
	Adult	1.36E-06	1.41E-10	1.60E-09	1.28E-09	5.46E-12	8.86E-09	5.01E-12	1.51E-10	3.06E-11	3.01E-08	1.24E-10	8.19E-10
	Child	8.91E-06	9.23E-10	1.05E-08	8.44E-09	3.57E-11	5.80E-08	3.28E-11	9.90E-10	2.01E-10	1.97E-07	8.16E-10	5.36E-09
	Inhalation												
	Adult	5.85E-09	1.82E-11	2.07E-10	1.66E-10	7.05E-13	1.14E-09	6.47E-13	1.95E-11	3.95E-12	3.88E-09	1.60E-11	1.05E-10
	Child	2.73E-08	8.49E-11	9.66E-10	7.77E-10	3.29E-12	5.34E-09	3.01E-12	9.11E-11	1.84E-11	1.81E-08	7.51E-11	4.94E-10
**A4**	Ingestion												
	Adult	4.89E-06	6.68E-08	1.47E-06	2.61E-06	1.91E-09	6.01E-06	4.56E-09	7.41E-07	6.68E-08	2.24E-05	6.71E-08	9.58E-08
	Child	2.28E-05	3.11E-07	6.90E-06	1.22E-05	8.94E-09	2.80E-05	2.13E-08	3.46E-06	3.11E-07	1.04E-04	3.13E-07	4.47E-07
	Dermal												
	Adult	5.86E-07	2.66E-10	5.90E-09	1.04E-08	7.65E-12	2.40E-08	1.82E-11	2.95E-09	2.66E-10	8.93E-08	2.68E-10	3.82E-10
	Child	3.84E-06	1.74E-09	3.86E-08	6.84E-08	5.01E-11	1.57E-07	1.19E-10	1.93E-08	1.74E-09	5.85E-07	1.75E-09	2.50E-09
	Inhalation												
	Adult	2.52E-09	3.44E-11	7.62E-10	1.34E-09	9.88E-13	3.10E-09	2.35E-12	3.82E-10	3.44E-11	1.15E-08	3.46E-11	4.94E-11

	Child	1.17E-08	1.60E-10	3.55E-09	6.29E-09	4.61E-12	1.44E-08	1.09E-11	1.78E-09	1.60E-10	5.38E-08	1.61E-10	2.30E-10
	Ingestion												
	Adult	2.58E-05	6.27E-08	1.14E-07	1.7E-07	8.21E-10	7.12E-08	1.20E-08	2.47E-07	7.31E-08	6.84E-06	7.30E-07	1.0E-06
	Child	1.21E-04	2.92E-07	5.32E-07	8.01E-07	3.83E-09	3.32E-07	5.63E-08	1.15E-06	3.41E-07	3.19E-05	3.40E-06	4.77E-06
	Dermal												
**B1**	Adult	3.09E-06	2.50E-10	4.55E-10	6.85E-10	3.27E-12	2.84E-10	4.81E-11	9.87E-10	2.91E-10	2.73E-08	2.91E-09	4.07E-09
	Child	2.02E-05	1.63E-09	2.98E-09	4.49E-09	2.14E-11	1.86E-09	3.15E-10	6.46E-09	1.91E-09	1.78E-07	1.90E-08	2.67E-08
	Inhalation												
	Adult	1.33E-08	3.23E-11	5.88E-11	8.85E-11	4.23E-13	3.67E-11	6.22E-12	1.27E-10	3.76E-11	3.52E-09	3.76E-10	5.26E-10
	Child	6.21E-08	1.50E-10	2.74E-10	4.13E-10	1.97E-12	1.71E-10	2.90E-11	5.95E-10	1.75E-10	1.64E-08	1.75E-09	2.45E-09
	Ingestion												
	Adult	5.18E-06	1.06E-07	2.69E-06	1.74E-07	1.64E-09	1.06E-07	3.6E-09	1.69E-07	1.43E-08	2.82E-05	7.90E-08	2.34E-08
	Child	2.42E-05	4.97E-07	1.25E-05	8.16E-07	7.67E-09	4.98E-07	1.71E-08	7.92E-07	6.68E-08	1.32E-04	3.68E-07	1.09E-07
	Dermal												
**B2**	Adult	6.20E-07	4.25E-10	1.07E-08	6.98E-10	6.55E-12	4.26E-10	1.46E-11	6.77E-10	5.71E-11	1.12E-07	3.15E-10	9.36E-11
	Child	4.06E-06	2.78E-09	7.04E-08	4.57E-09	4.29E-11	2.79E-09	9.60E-11	4.43E-09	3.74E-10	7.38E-07	2.06E-09	6.13E-10
	Inhalation												
	Adult	2.67E-09	5.49E-11	1.38E-09	9.01E-11	8.47E-13	5.50E-11	1.89E-12	8.75E-11	7.38E-12	1.45E-08	4.07E-11	1.21E-11
	Child	1.24E-08	2.56E-10	6.47E-09	4.20E-10	3.95E-12	2.56E-10	8.83E-12	4.08E-10	3.44E-11	6.79E-08	1.90E-10	5.64E-11

B3	Ingestion												
	Adult	3.39E-06	1.06E-07	4.12E-06	2.72E-07	1.23E-08	2.51E-06	5.79E-09	2.23E-08	8.33E-08	1.12E-04	5.82E-08	1.27E-07
	Child	1.58E-05	5.62E-08	1.92E-05	1.27E-06	5.75E-08	1.17E-05	2.70E-08	1.04E-07	3.89E-07	5.24E-04	2.72E-07	5.94E-07
	Dermal												
	Adult	4.06E-07	4.80E-11	1.64E-08	1.08E-09	4.91E-11	1.00E-08	2.31E-11	8.92E-11	3.32E-10	4.48E-07	2.32E-10	5.08E-10
	Child	2.66E-06	3.15E-10	1.07E-07	7.12E-09	3.22E-10	6.56E-08	1.51E-10	5.84E-10	2.17E-09	2.93E-06	1.52E-09	3.32E-09
	Inhalation												
	Adult	1.75E-09	6.21E-12	2.12E-09	1.40E-10	6.35E-12	1.29E-09	2.98E-12	1.15E-11	4.29E-11	5.78E-08	3.00E-11	6.56E-11
	Child	8.16E-09	2.89E-11	9.92E-09	6.56E-10	2.96E-11	6.04E-09	1.39E-11	5.38E-11	2.00E-10	2.70E-07	1.40E-10	3.06E-10
B4	Ingestion												
	Adult	1.04E-05	2.38E-08	4.57E-06	1.18E-07	3.56E-09	1.06E-07	1.89E-09	6.84E-09	9.92E-08	6.16E-06	3.71E-08	4.60E-07
	Child	4.87E-05	1.11E-07	2.13E-05	5.55E-07	1.66E-08	4.98E-07	8.84E-09	3.19E-08	4.63E-07	2.87E-05	1.73E-07	2.14E-06
	Dermal												
	Adult	1.25E-06	9.51E-11	1.82E-08	4.74E-10	1.42E-11	4.26E-10	7.56E-12	2.73E-11	3.96E-10	2.45E-08	1.48E-10	1.83E-09
	Child	8.19E-06	6.22E-10	1.19E-07	3.10E-09	9.30E-11	2.79E-09	4.95E-11	1.78E-10	2.59E-09	1.60E-07	9.70E-10	1.20E-08
	Inhalation												
	Adult	5.38E-09	1.22E-11	2.35E-09	6.13E-11	1.83E-12	5.50E-11	9.76E-13	3.52E-12	5.11E-11	3.17E-09	1.91E-11	2.37E-10
	Child	2.51E-08	5.73E-11	1.10E-08	2.86E-10	8.56E-12	2.56E-10	4.55E-12	1.64E-11	2.38E-10	1.48E-08	8.92E-11	1.10E-09
C	Ingestion												

Adult	1.56E-05	5.89E-08	1.3E-06	1.09E-06	7.56E-08	6.08E-06	3.08E-09	7.39E-08	2.72E-08	3.10E-05	8.0E-08	4.99E-07
Child	7.28E-05	2.74E-07	6.24E-06	5.12E-06	3.52E-07	2.83E-05	1.43E-08	3.45E-07	1.27E-07	1.45E-04	3.75E-07	2.32E-06
Dermal												
Adult	1.86E-06	2.35E-10	5.33E-09	4.38E-09	3.01E-10	2.42E-08	1.22E-11	2.95E-10	1.08E-10	1.24E-07	3.20E-10	1.99E-09
Child	1.22E-05	1.53E-09	3.49E-08	2.87E-08	1.97E-09	1.58E-07	8.05E-11	1.93E-09	7.12E-10	8.12E-07	2.10E-09	1.30E-08
Inhalation												
Adult	8.04E-09	3.03E-11	6.89E-10	5.66E-10	3.89E-11	3.13E-09	1.58E-12	3.81E-11	1.40E-11	1.60E-08	4.14E-11	2.57E-10
Child	3.75E-08	1.41E-10	3.21E-09	2.64E-09	1.81E-10	1.46E-08	7.41E-12	1.77E-10	6.55E-11	7.47E-08	1.93E-10	1.20E-09
