# Supplementary material for: Investigative health and ecological risk assessment of trace elements in pharmaceutical deposition near Dhaka: An endemic industrial surge of Bangladesh
Source: PLoS One. 2026 Jan 5;21(1):e0338816. doi: 10.1371/journal.pone.0338816 (PMC12768289; doi:10.1371/journal.pone.0338816)
Supplement: S5 Table — (PDF) [file pone.0338816.s005.pdf]

S5 Table: Physicochemical Parameters (Mean ± SD) of water samples

**Sample site**	**pH**	**TDS (mg/L)**	**EC (µS/cm)**	**COD (mg/L)**	**DO (mg/L)**	**Temp (º C)**
A1	8.48 ± 0.02	427.34 ± 0.09	854.52 ± 0.02	31.2 ± 0.1	4.91 ± 0.09	26.5 ± 0.1
A2	8.43 ± 0.01	456.19 ± 0.05	913.26 ± 0.01	51.9 ± 0.3	7.98 ± 0.07	27.1 ± 0.2
A3	8.17 ± 0.04	131.80 ± 0.02	263.76 ± 0.02	9.1 ± 0.4	6.65 ± 0.03	26.5 ± 0.1
A4	8.21 ± 0.02	416.55 ± 0.11	826.98 ± 0.05	73.6 ±0.1	2.15 ± 0.04	28.3 ± 0.1
B1	8.57 ± 0.03	431.43 ± 0.05	863.91 ± 0.04	52.1 ± 0.2	4.93 ± 0.05	27.3 ± 0.2
B2	8.23 ± 0.01	596.24 ± 0.08	1201.42 ± 0.03	64.3 ± 0.3	2.02 ± 0.03	27.6 ±0.1
B3	8.39 ± 0.04	235.29 ± 0.10	469.50 ± 0.01	23.3 ± 0.5	3.55 ± 0.07	24.8 ± 0.2
B4	7.8 ± 0.01	105.46 ± 0.09	211.04 ± 0.01	4.2 ± 0.1	3.64 ± 0.01	25.9 ± 0.3
C	7.96 ± 0.03	295.05 ± 0.02	590.96 ± 0.04	6.1 ± 0.4	7.03 ± 0.02	26.8 ± 0.1
ECR	6.5-8.5	1000	2250	100	≥ 5	25

SD = Standard deviation
