# Supplementary material for: Investigative health and ecological risk assessment of trace elements in pharmaceutical deposition near Dhaka: An endemic industrial surge of Bangladesh
Source: PLoS One. 2026 Jan 5;21(1):e0338816. doi: 10.1371/journal.pone.0338816 (PMC12768289; doi:10.1371/journal.pone.0338816)
Supplement: S7 Table — (PDF) [file pone.0338816.s007.pdf]

S7 Table: PCA of two components with the respective loading of metals of the study area

	Water			Sediment		
	PC 1	PC 2	PC 3	PC 1	PC 2	PC 3
As	0.467	0.051	-0.006	0.339	-0.377	0.162
Se	0.068	0.270	-0.359	0.379	-0.328	0.005
Pb	-0.146	0.526	0.215	0.336	0.138	0.444
Be	-0.035	0.177	0.039	0.254	-0.434	0.136
Cd	-0.186	0.453	0.235	-0.051	0.488	0.235
Co	0.383	0.010	0.242	0.261	0.101	-0.296
Cr	0.023	0.473	0.240	0.292	0.260	-0.246
Cu	0.112	-0.124	0.612	0.319	0.119	-0.410
Mn	-0.191	-0.175	0.445	0.317	0.155	-0.445
Ni	0.490	0.027	0.102	0.355	0.250	0.314
V	0.468	-0.032	0.071	0.253	0.100	0.248
Hg	-0.249	-0.371	0.247	0.108	0.337	0.161
Eigen value	3.875	2.785	1.911	4.161	2.860	2.285
% Total variance	32.30%	23.21%	15.93%	34.77%	23.84%	19.04%
Cumulative %	32.30%	55.51%	71.44%	34.77%	58.61%	77.65%
