# Supplementary material for: Investigative health and ecological risk assessment of trace elements in pharmaceutical deposition near Dhaka: An endemic industrial surge of Bangladesh
Source: PLoS One. 2026 Jan 5;21(1):e0338816. doi: 10.1371/journal.pone.0338816 (PMC12768289; doi:10.1371/journal.pone.0338816)
Supplement: S1 File — (PDF) [file pone.0338816.s008.pdf]

# Supporting information about the Method

**Water pollution status and health risk assessment of metals in water**

**Heavy metal pollution index (HPI)**

HPI was used to determine a rating or weightage (Wi) for each metal constituent. HPI was calculated according to the following equation: (Rahman et al., 2020)

$$HPI = \frac{\sum_{i=1}^{n} Q_i W_i}{\sum_{i=1}^{n} W_i}$$

Where, $W_i$ = the mass ratio of trace heavy metal number i; $Q_i$= the quality index of heavy metal number i; n = total number of monitoring heavy metal.

**Heavy metal evaluation index (HEI)**

HEI describes water quality condition in response to anthropogenic heavy metals and was calculated by the following equation (Rahman et al., 2020):

$$\mathrm{HEI} = \sum_{\mathrm{i=1}}^{\mathrm{n}} \frac{H_c}{H_{mac}}$$

$H_c$ is observed concentration of heavy metal, $H_{mac}$ is maximum admissible concentration of the ith parameter.

**Contamination index/ Degree of contamination ($C_d$)**

The contamination index ($C_d$) describes the quality of water which is being evaluated based on the degree of contamination. The $C_d$ was computed separately for each sample of water analyzed, as a sum of the contamination factors of individual components exceeding the upper permissible value. Hence the $C_d$ summarizes the combined effects of several quality parameters which are considered harmful to household water. The contamination index was calculated using the equation shown below:

$$\mathrm{C_d} = \sum_{\mathrm{i=1}}^{\mathrm{n}} C_{fi} \text{ and } C_{fi} = \frac{C_{Ai}}{C_{Ni}} - 1$$

where, $C_{fi}$ = contamination factor for the ith component, $C_A$ = analytical value for the ith component, $C_{Ni}$ = upper permissible concentration of the ith component (N denotes the 'normative value'). The resultant $C_d$ values are grouped into three categories which are as follows: $C_d < 1$ (low), $C_d$ = 1-3 (medium) and $C_d > 3$ (high) (Zakir et al., 2020).

**Hazard Quotient and Hazard Index**

The USEPA investigates a variety of scientific techniques for evaluating danger while using contaminated water. In this study, drinking polluted water (ingestion), inhalation and skin contact (dermal) as routes of element exposure to the human body were primarily taken into account. As a result, reference doses and the chronic daily intake (CDI), which were computed for both adults and children based on USEPA regulations, were compared to the investigated elements. The following equations were used to compute the expected doses of exposure through ingestion, dermal and inhalation routes, respectively (Hasan et al., 2021).

$$CDI_{ingestion} = \frac{EC \times IngR \times EF \times ED}{BW \times AT}$$

$$CDI_{dermal} = \frac{EC \times SA \times AF \times ABS \times ET \times EF \times ED \times CF}{BW \times AT}$$

$$CDI_{inhalation} = \frac{Inh\ R \times EF \times ED}{BW \times AT \times PEF}$$

where, EC = concentration of the metal (mg/L), Ing R = ingestion rate (l $day^{-1}$) (for adult: 2.5, child: 0.78) (USEPA, 1989), Inh R = inhalation rate ($m^3$ $day^{-1}$) (USEPA, 1989), EF =the exposure frequency (day $year^{-1}$) (365) (USEPA, 1989), ED = the exposure duration (year) (for adult: 70, child: 6) (USEPA, 2002), BW = BW is the bodyweight (kg) (for adult: 70, child: 15) (USEPA, 1991), AT = the average time (day) (ED × 365) (USEPA, 1989), PEF = particle emission factor ($1.32 \times 10^9$ $m^3$/kg) (USEPA, 2011), SA = the exposed skin area ($cm^2$) (5700) (USEPA, 2011), AF = the adherence factor (mg $cm^2$) (0.07) (USEPA, 2011), $ABS_d$ = the dermal absorption fraction (0.03) (USEPA, 2011), ET = the exposure time (0.6 (hour $day^{-1}$) (USEPA, 2002), CF = the conversion factor (kg $mg^{-1}$) ($10^{-6}$) (USEPA, 2002). In order to determine the hazard quotient (HQ) and hazard index (HI) for the human population associated with the metal contamination of water, the following equations were used (USEPA, 1989, USEPA, 2002)-

$$HQ_{ingestion} = \frac{CDI_{ingestion}}{RfD_{ingestion}}$$

$$HQ_{dermal} = \frac{CDI_{dermal}}{RfD_{dermal}}$$

$$HQ_{inhalation} = \frac{CDI_{inhalation}}{RfD_{inhalation}}$$

$$HI = \sum HQ_i$$

where, RfD ingestion and RfD dermal are the reference dose for oral and dermal exposures, in mg/kg/day, based on USEPA. Risk-based concentrations for all metals were obtained from USEPA standards (USEPA, 2011, USEPA, 2010, USEPA, 2006) except Pb, which was derived from the guidelines of the World Health Organization (WHO, 2006). The RfD ingestion and RfD dermal values are as follows: Mn-0.02, Cu-0.04, Zn-0.3, Ni-0.02, Cd-0.0005, V-0.001, Cr- 0.003, and Pb-0.0014 for RfD ingestion and Mn-0.0008, Cu-0.012, Zn-0.06, Ni-0.0054, Cd-0.000005, Cr-0.000015, Co-0.06, and Pb-0.00042 for RfD dermal. The HI and HQ values greater than 1, denotes a prolonged high risk to human health (Hasan et al., 2021).

**Carcinogenic Risks**

Multiplying CDI values by the cancer slope factor (SF), the probability of developing cancer using polluted water during a lifetime was calculated (Hasan et al., 2021). The carcinogenic risk was determined both for an individual metal ($Risk_i$) and multiple metals (Risk total), which were calculated by the following equations respectively:

$$Carcinogenic\ Risk_j = CDI \times SF$$

$$Carcinagenic\ Risk\ Total = \sum_{i=1}^{m} \sum_{j=1}^{n} Risk_{ij}$$

where, carcinogenic risk for an individual expresses the probability during the lifetime of an individual exposed to carcinogens, and carcinogenic risk total is the sum of cancer risk for heavy metals i in exposure pathway j. SF (mg $kg^{-1}$ $day^{-1}$) represents the cancer slope factor. As, Cd, and Cr are identified as human carcinogens and Pb is categorized as a probable human carcinogen by the International Agency for Research on Cancer (Zhang et al., 2015). The applied slope factors were as follows: 1.5 for As, 0.0085 for Pb, 0.5 for Cr,15 for Cd and 1.7 for Ni. For managing the risk, the cancer risk from $1 \times 10^{-6}$ to $1 \times 10^{-4}$ is considered acceptable or tolerable (Zhang et al., 2015).

**Geoaccumulation index ($I_{geo}$)**

The geoaccumulation index ($I_{geo}$), a widely used method for assessing soil and sediment contaminations, can be used to determine the extent of trace metal contamination in sediment at the preliminary stage (Islam et al., 2015) and was calculated by the following equation:

$$I_{geo} = \log_2\left(\frac{C_n}{1.5B_n}\right)$$

where $C_n$ is the measured concentration of the metal 'n' in the soil and $B_n$ is the geochemical background value in sediments (average shale) (Turekian and Wedepohl, 1961). The $B_n$ of As, Se, Pb, Be, Cd, Co, Cr, Cu, Mn, Ni, V and Hg was assumed 13, 0.6, 20, 3, 0.3, 19, 90, 45, 850, 68, 130 and 0.4 respectively according to Turekian and Wedepohl (Turekian and Wedepohl, 1961). The $I_{geo}$ values were categorized in seven classes as bellow (Müller, 1981)-

$I_{geo}$: ≤ 0 means practically uncontaminated; 0 to 1 means uncontaminated to moderately contaminated; 1 to 2 indicates moderately contaminated; 2 to 3indicates moderately to heavily contaminated; 3 to 4 means heavily contaminated; 4 to 5 means heavily to extremely contaminated; >5 indicates extremely contaminated.

**Enrichment Factor (EF)**

Enrichment Factor (EF) is a frequently used measure to assess the level of environmental toxicity and distinguish between natural and anthropogenic sources of heavy metals (Hu et al., 2013). The analyzed metal concentrations of

sediment samples were first normalized relative to reference elements (iron, aluminium or manganese) to evaluate if a sediment sample is enriched with metals relative to the sample's background conditions (Rahman et al., 2022). Manganese was selected as the normalizing element in this study. The following equation was used to calculate the EF-

$$EF = \frac{\left(\frac{C_x}{C_{Mn}}\right)_{sample}}{\left(\frac{C_x}{C_{Mn}}\right)_{background}}$$

Here, $\left(\frac{C_x}{C_{Mn}}\right)_{sample}$ is the ratio of the concentration of the respective element ($C_x$) to that of Mn ($C_{Mn}$) in the sediment sample, $\left(\frac{C_x}{C_{Mn}}\right)_{background}$ is the background ratio of the same metal.

Soil contamination according to EF value was differentiated in the ways (Taylor, 1964)-

EF< 1 indicates no enrichment; 1 < EF < 3, minor enrichment; 3 < EF < 5, moderate enrichment; 5 < EF < 10, moderately severe enrichment; 10 < EF < 25, severe enrichment; 10 < EF < 25, very severe enrichment and EF > 50 means extremely severe enrichment. Besides, EF > 1.5 indicates that these metals might be from non-crustal materials or non-natural weathering processes (Gao and Chen, 2012).

**Contamination Factor (CF) and Pollution load index (PLI)**

The contamination factor (CF) stands for the ratio of the measured concentration of each element to the natural abundance of the same metal (Rahman et al., 2022).

$$CF = \frac{(Metal\ concentration)_{sample}}{(Metal\ concentration)_{background}}$$

CF values are categorized in to four groups (Hakanson, 1980)- CF < 1, low contamination, 1 ≤ CF < 3, moderate contamination; 3 ≤ CF < 6, considerable contamination; CF ≥ 6, very high contamination.

The pollution load index (PLI) is an integrated approach to determine whether a sampling site is polluted or non-polluted by metals.

$$PLI = (CF_1 \times CF_2 \times CF_3 \times \ldots\ldots\ldots\ldots\ldots CF_n)^{1/n}$$

where n is the number of metals and CF is the contamination factor. The pollution load index provides a simple, comparative means for assessing the level of metal pollution and classified as (Mashiatullah et al., 2013)- PLI< 1, no pollution; 1 < PLI < 2, moderate pollution; 2 < PLI < 3, heavy pollution; PLI > 3, extremely heavy pollution.

**Potential Ecological Risk (PER)**

A potential ecological risk index (PER) expresses the pollution level of heavy metals in sediments. The equation used to calculate PER according to Luo et al.2007 is as follows (Luo et al., 2007):

$$E_r^i = T_r^i \times C_r^i \text{ and } PER = \sum_{i=1}^{m} E_r^i$$

where, $E^i_r$ = the potential ecological risk factor, $C^i_r$ = single element contamination factor, $T^i_r$ = biological toxic factor of individual metal. In this study, biological toxic factor for As, Pb, Cd, Co, Cr, Cu, Mn, Ni, Zn and Hg were used as 10, 5, 40, 1, 2, 5, 1, 5, 1, 40 respectively (Hakanson, 1980). PER is classified into four groups (Ahamad et al., 2020)- PER< 110 means low ecological risk; 110 ≤ PER < 200 means moderate risk; 200 ≤ PER < 400, considerable risk and PER ≥ 400 defines very high risk.

\

**References**


AHAMAD, M. I., SONG, J., SUN, H., WANG, X., MEHMOOD, M. S., SAJID, M., SU, P. & KHAN, A. J. 2020. Contamination Level, Ecological Risk, and Source Identification of Heavy Metals in the Hyporheic Zone of the Weihe River, China. *Int J Environ Res Public Health,* 17.

GAO, X. & CHEN, C. T. 2012. Heavy metal pollution status in surface sediments of the coastal Bohai Bay. *Water Res,* 46**,** 1901-11.

HAKANSON, L. 1980. An ecological risk index for aquatic pollution control.a sedimentological approach. *Water Research,* 14**,** 975-1001.

HASAN, M. F., NUR-E-ALAM, M., SALAM, M. A., RAHMAN, H., PAUL, S. C., RAK, A. E., AMBADE, B. & TOWFIQUL ISLAM, A. R. M. 2021. Health Risk and Water Quality Assessment of Surface Water in an Urban River of Bangladesh. *Sustainability,* 13**,** 6832.

HU, B., CUI, R., LI, J., WEI, H., ZHAO, J., BAI, F., SONG, W. & DING, X. 2013. Occurrence and distribution of heavy metals in surface sediments of the Changhua River Estuary and adjacent shelf (Hainan Island). *Marine Pollution Bulletin,* 76**,** 400-405.

ISLAM, M. S., AHMED, M. K., RAKNUZZAMAN, M., HABIBULLAH -AL- MAMUN, M. & ISLAM, M. K. 2015. Heavy metal pollution in surface water and sediment: A preliminary assessment of an urban river in a developing country. *Ecological Indicators,* 48**,** 282-291.

LUO, W., LU, Y., GIESY, J. P., WANG, T., SHI, Y., WANG, G. & XING, Y. 2007. Effects of land use on concentrations of metals in surface soils and ecological risk around Guanting Reservoir, China. *Environmental Geochemistry and Health,* 29**,** 459-471.

MASHIATULLAH, A., CHAUDHARY, M. Z., AHMAD, N., JAVED, T. & GHAFFAR, A. 2013. Metal pollution and ecological risk assessment in marine sediments of Karachi Coast, Pakistan. *Environmental Monitoring and Assessment,* 185**,** 1555-1565.

MÜLLER, G. 1981. The Heavy Metal Pollution of the Sediments of Neckars and its Tributary: A Stocktaking. *Chemiker Zeitung,* 105**,** 157-164.

RAHMAN, M., SAIMA, J., RIMA, S. A., HOSSAIN, M. I. S., DAS, D. K., BAKAR, M. A. & SIDDIQUE, M. A. M. 2022. Ecological risks of heavy metals on surficial sediment of Nijhum Dweep (Island), an important biodiversity area of Bangladesh. *Marine Pollution Bulletin,* 179**,** 113688.

RAHMAN, M. A. T. M. T., PAUL, M., BHOUMIK, N., HASSAN, M., ALAM, M. K. & AKTAR, Z. 2020. Heavy metal pollution assessment in the groundwater of the Meghna Ghat industrial area, Bangladesh, by using water pollution indices approach. *Applied Water Science,* 10**,** 186.

TAYLOR, S. R. 1964. Abundance of chemical elements in the continental crust: a new table. *Geochimica et Cosmochimica Acta,* 28**,** 1273-1285.

TUREKIAN, K. K. & WEDEPOHL, K. H. 1961. Distribution of the Elements in Some Major Units of the Earth's Crust. *GSA Bulletin,* 72**,** 175-192.

USEPA 1989. *Risk Assessment Guidance for Superfund, Volume I: Human Health Evaluation Manual (Part A); Office for Emergency and Remedial Response,* Washington DC, USA, US Environment Protection Agency.

USEPA 1991. *Human Health Evaluation Supplemental Guidance, Standard Default Exposure Factors* Washington DC, USA, US Environment Protection Agency.

USEPA 2002. *Supplemental Guidance for Developing Soil Screening Levels for Superfund Sites, Appendix D-Dispersion Factors Calculations,* Washington, DC, USA.

USEPA 2006. *ENERGY STAR Overview of 2006 Achivements,* Washington DC, US Environment Protection Agency.

USEPA 2010. *Intigrated Risk Information System (IRIS),* Washington DC, USA, US Environment Protection Agency.

USEPA 2011. *Exposure Factors Handbook,* Washington DC, USA, US Environment Protection Agency.

WHO 2006. *Guidelines for Drinking Water Quality,* Geneva, Switzerland, World Health Organization.

ZAKIR, H. M., SHARMIN, S., AKTER, A. & RAHMAN, M. S. 2020. Assessment of health risk of heavy metals and water quality indices for irrigation and drinking suitability of waters: a case study of Jamalpur Sadar area, Bangladesh. *Environmental Advances,* 2**,** 100005.

ZHANG, L., MO, Z., QIN, J., LI, Q., WEI, Y., MA, S., XIONG, Y., LIANG, G., QING, L., CHEN, Z., YANG, X., ZHANG, Z. & ZOU, Y. 2015. Change of water sources reduces health risks from heavy metals via ingestion of water, soil, and rice in a riverine area, South China. *Sci Total Environ,* 530-531**,** 163-170.
